# Supplementary material for: Novel artificial selection method improves function of simulated microbial communities
Source: PLoS Comput Biol. 2026 Jan 13;22(1):e1013863. doi: 10.1371/journal.pcbi.1013863 (PMC12829962; doi:10.1371/journal.pcbi.1013863)
Supplement: S4 Table — Parameters defining a microbial strain i in the ODE model. Growth and degradation parameters in relation to nutrients and toxic compounds Nj and Tk. All parameters are assumed to be positive, and the investment fik is limited to the interval [0, 1]. The matrices of growth rates, death rates and degradation investment rij, mik and fik are made sparse by multiplying them by matrices drawn from Bernoulli(0.5), i.e. flipping a coin for each entry. In this way, each species takes up approximately half of the nutrients, is affected by half of the toxic compounds and degrades half of the toxic compounds. (PDF) [file pcbi.1013863.s004.pdf]

Parameter	Description	Sampled from
l_i	Species ID of strain i	
r_{ij}	Maximum growth rate<br>with respect to nutrient j	Uni(0.01, 0.1)<br>Sparse
K_N	Half-saturation constant<br>for nutrients	$K_N = 10$<br>(fixed)
m_{ik}	Maximum death rate<br>with respect to toxic compound k	Uni(10^{-4}, 10^{-3})<br>Sparse
K_T	Half-saturation constant<br>for toxic compounds	$K_T = 10$<br>(fixed)
f_{ik}	Fraction of amassed nutrients<br>that are invested into<br>degradation of toxic compound k	Uni(0, 1), Sparse<br>Rescaled so that<br>$\sum_k f_{ik} = \mathrm{Uni}(0, 1)$
Y_i	(Average) biomass yield<br>with respect to the nutrients	lognormal(log(10^{-3}), log(5))
δ_i	(Average) degradation efficiency<br>with respect to the toxic compounds	lognormal(log(10^{-4}), log(5))

S4 Table Parameters defining a microbial strain i in the ODE model. Growth and degradation parameters in relation to nutrients and toxic compounds N_j and T_k. All parameters are assumed to be positive, and the investment f_{ik} is limited to the interval [0, 1]. The matrices of growth rates, death rates and degradation investment r_{ij}, m_{ik} and f_{ik} are made sparse by multiplying them by matrices drawn from Bernoulli(0.5), i.e. flipping a coin for each entry. In this way, each species takes up approximately half of the nutrients, is affected by half of the toxic compounds and degrades half of the toxic compounds.
