# Supplementary material for: Novel artificial selection method improves function of simulated microbial communities
Source: PLoS Comput Biol. 2026 Jan 13;22(1):e1013863. doi: 10.1371/journal.pcbi.1013863 (PMC12829962; doi:10.1371/journal.pcbi.1013863)
Supplement: S1 Fig — Illustration of the variables and processes in the ODE model. Populations of cells vary in their preferences for nutrients and degradation capabilities. The populations use the available nutrients to degrade the toxic compounds and to grow. (PDF) [file pcbi.1013863.s005.pdf]

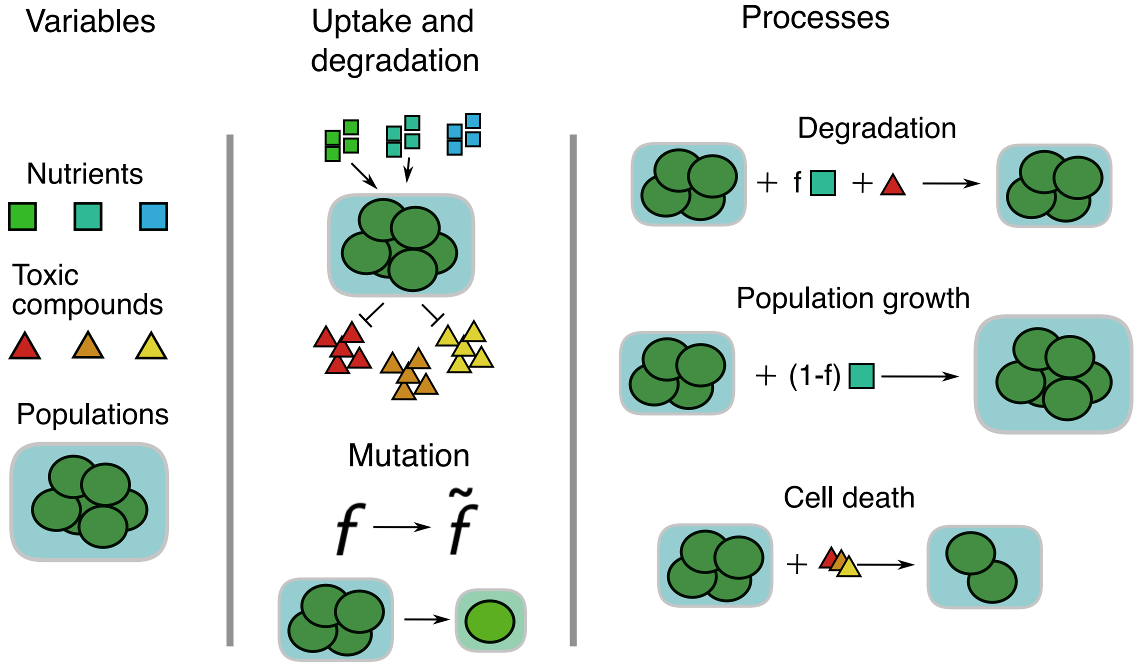
Variables
Uptake and degradation
Processes
Nutrients
Toxic compounds
Populations
Mutation
$f \longrightarrow \tilde{f}$
Degradation
+ f
+
Population growth
+ (1-f)
Cell death
+
