# Supplementary figures and images for: Novel artificial selection method improves function of simulated microbial communities
Source: PLoS Comput Biol. 2026 Jan 13;22(1):e1013863. doi: 10.1371/journal.pcbi.1013863 (PMC12829962; doi:10.1371/journal.pcbi.1013863)

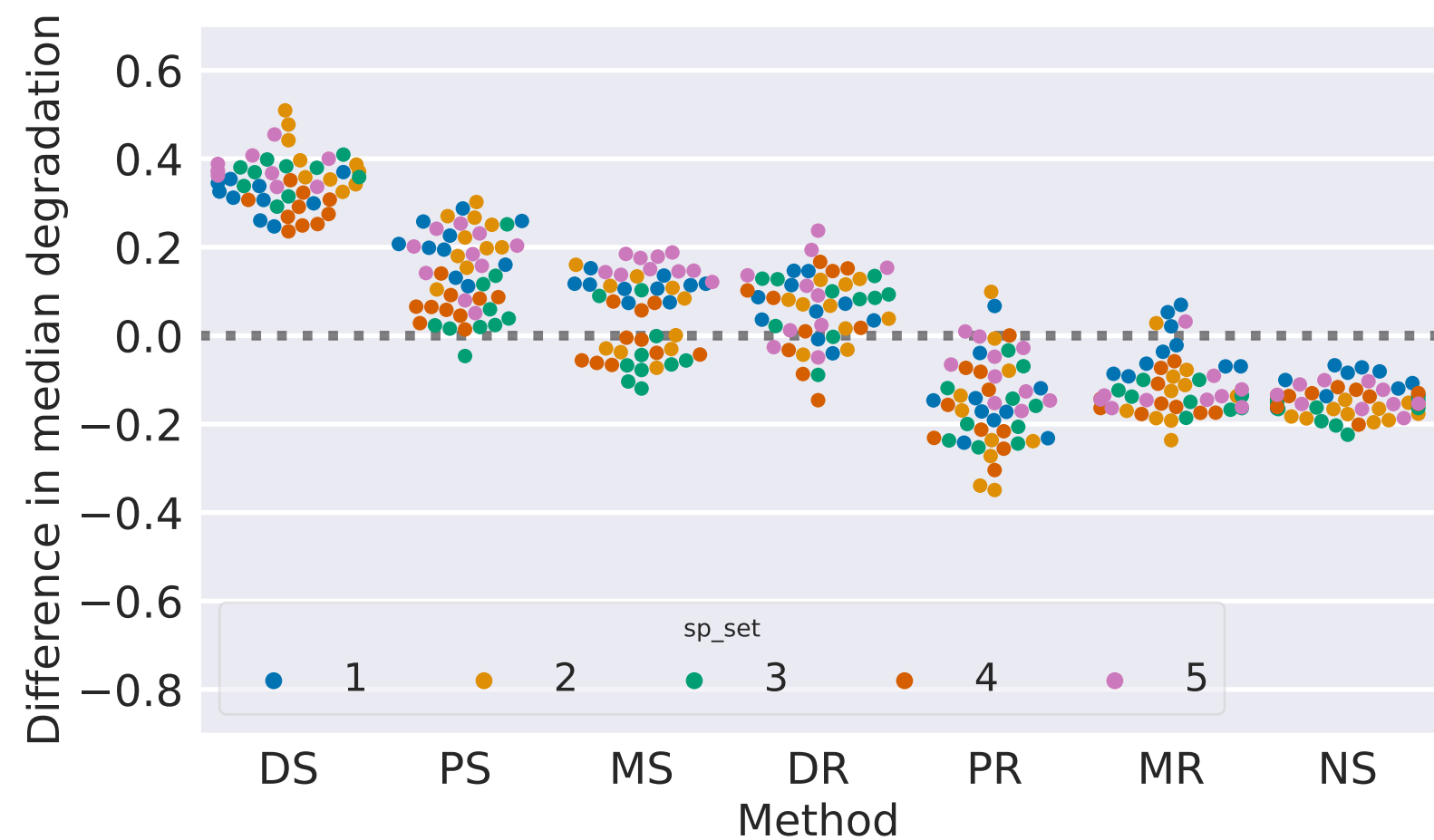

Supplement: S2 Fig — The difference in median degradation between round 50 and round 0 for each propagation method, corresponding to Fig 2 where the difference in maximum degradation is shown. The two-sided Wilcoxon test for difference in degradation against the no-selection control is significant for the selection methods DS, PS and MS. Data generated by the IBM. (PDF) [file pcbi.1013863.s006.pdf]

A

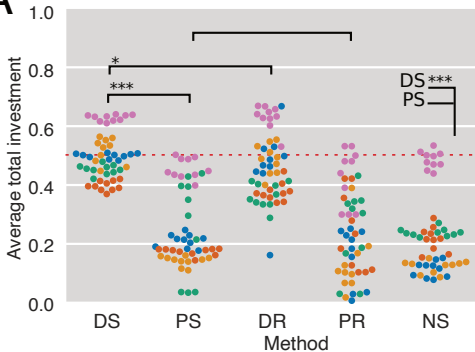

B

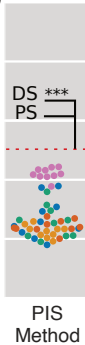

Supplement: S4 Fig — Average total investment into degradation of communities at round 50, averaged for the 21 communities in a run, corresponding to panel A, B in Fig 3, but generated by the ODE. (PDF) [file pcbi.1013863.s008.pdf]

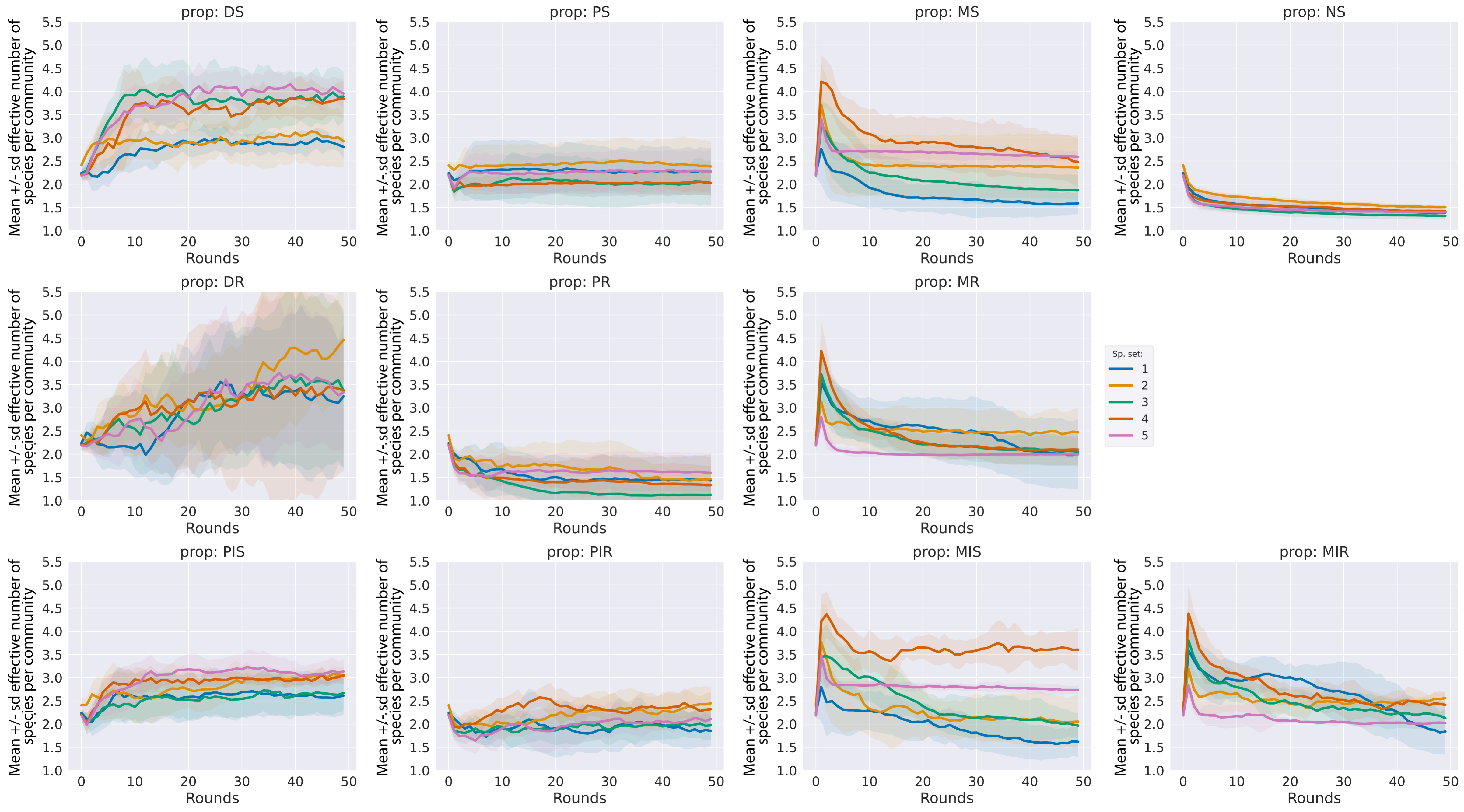

Supplement: S5 Fig — Time-series of the species diversity (effective number of species per community) corresponding to Fig 3C. Each panel shows the mean ± standard deviation over the 10 repeated runs, for each species set 1-5, for one propagation method. Data generated by the IBM. (PDF) [file pcbi.1013863.s009.pdf]

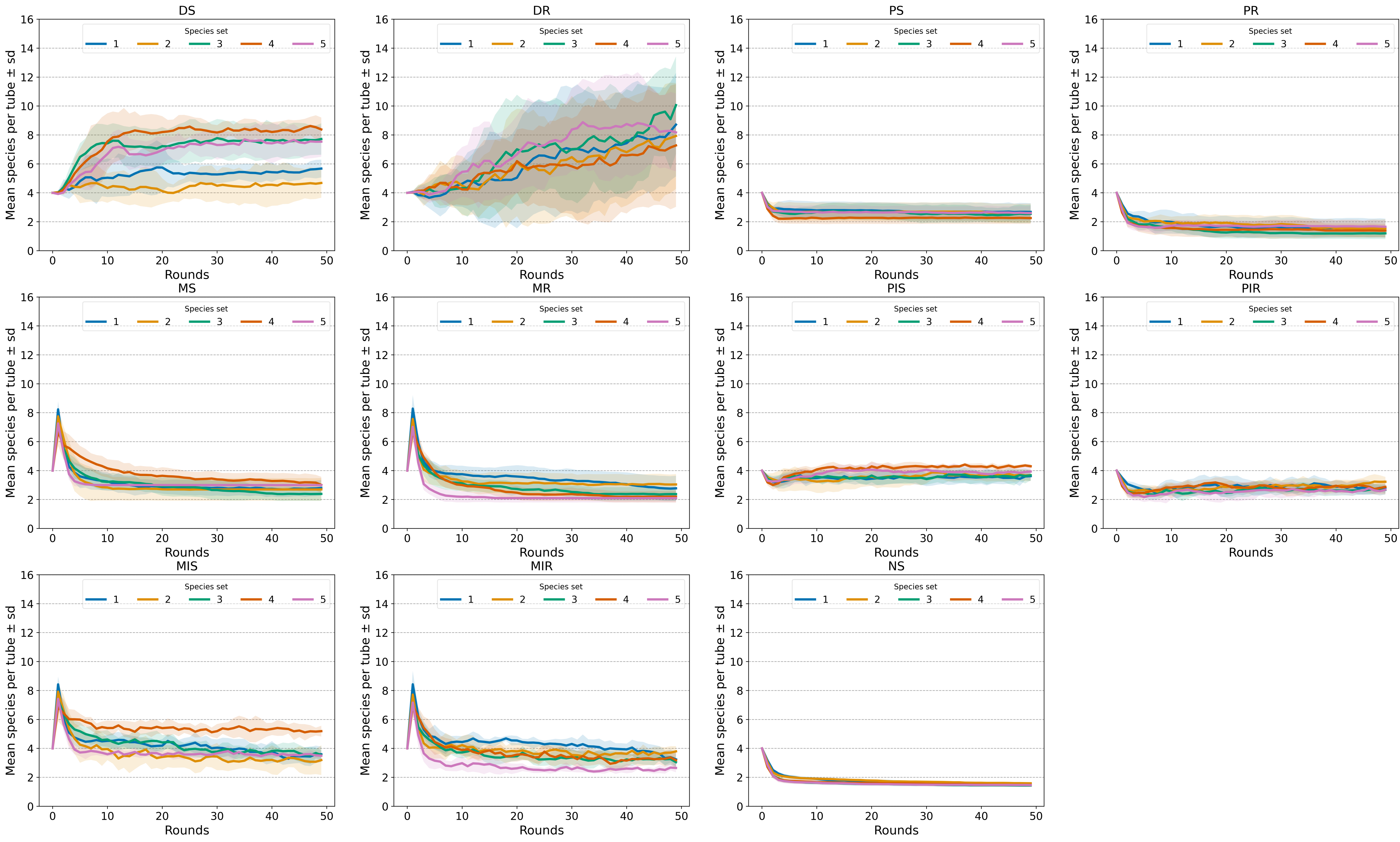

Supplement: S6 Fig — Time-series of the mean number of species per community (richness) corresponding to Fig 3C. This includes all communities in a round. Each panel shows the mean ± standard deviation over the 10 repeated runs, for each species set 1-5, for one propagation method. Data generated by the IBM. (PDF) [file pcbi.1013863.s010.pdf]

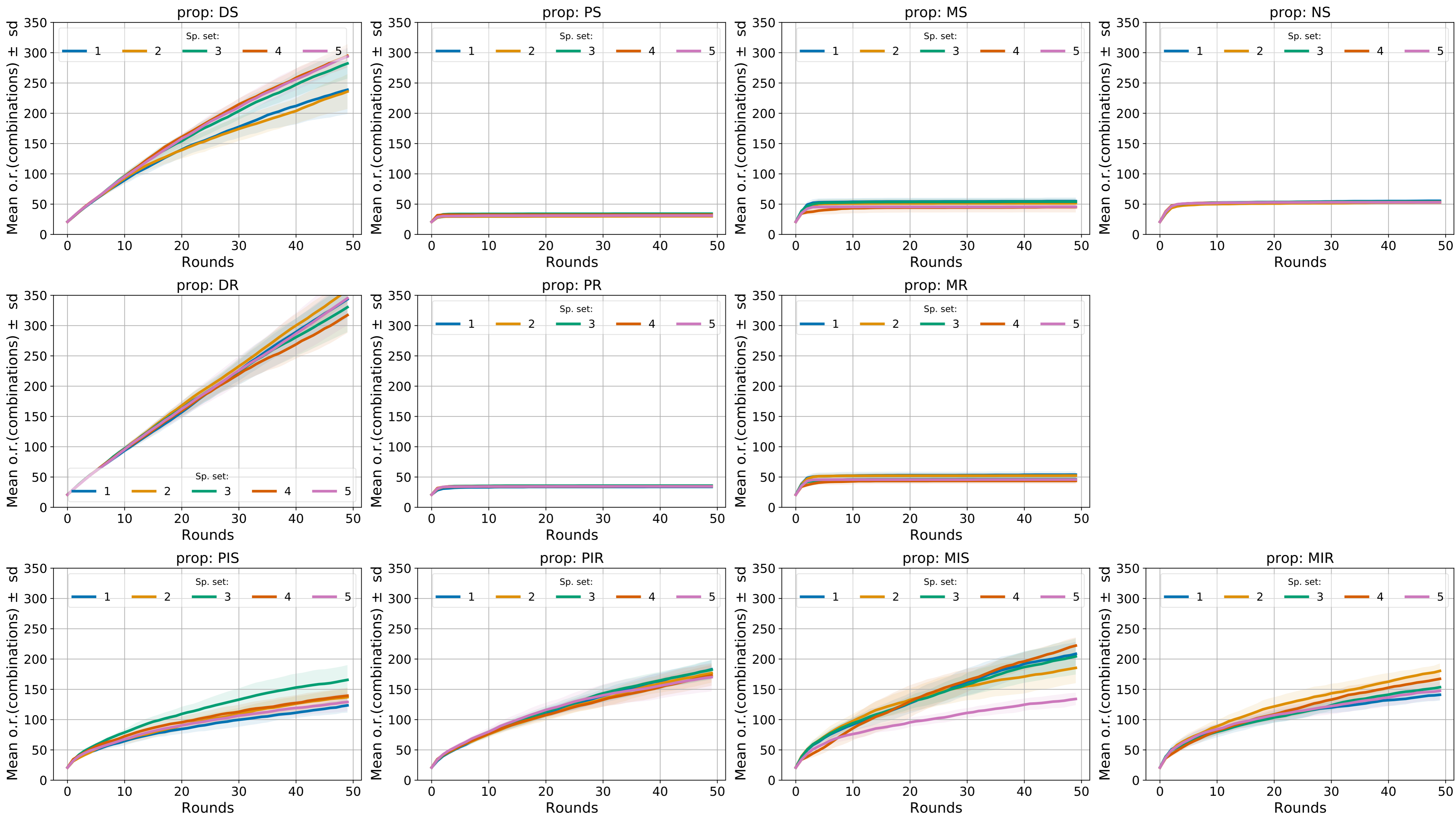

Supplement: S7 Fig — Time-series of the number of explored communities, corresponding to Fig 4A. Each panel shows the mean ± standard deviation over the 10 repeated runs, for each species set 1-5, for one propagation method. Data generated by the IBM. (PDF) [file pcbi.1013863.s011.pdf]

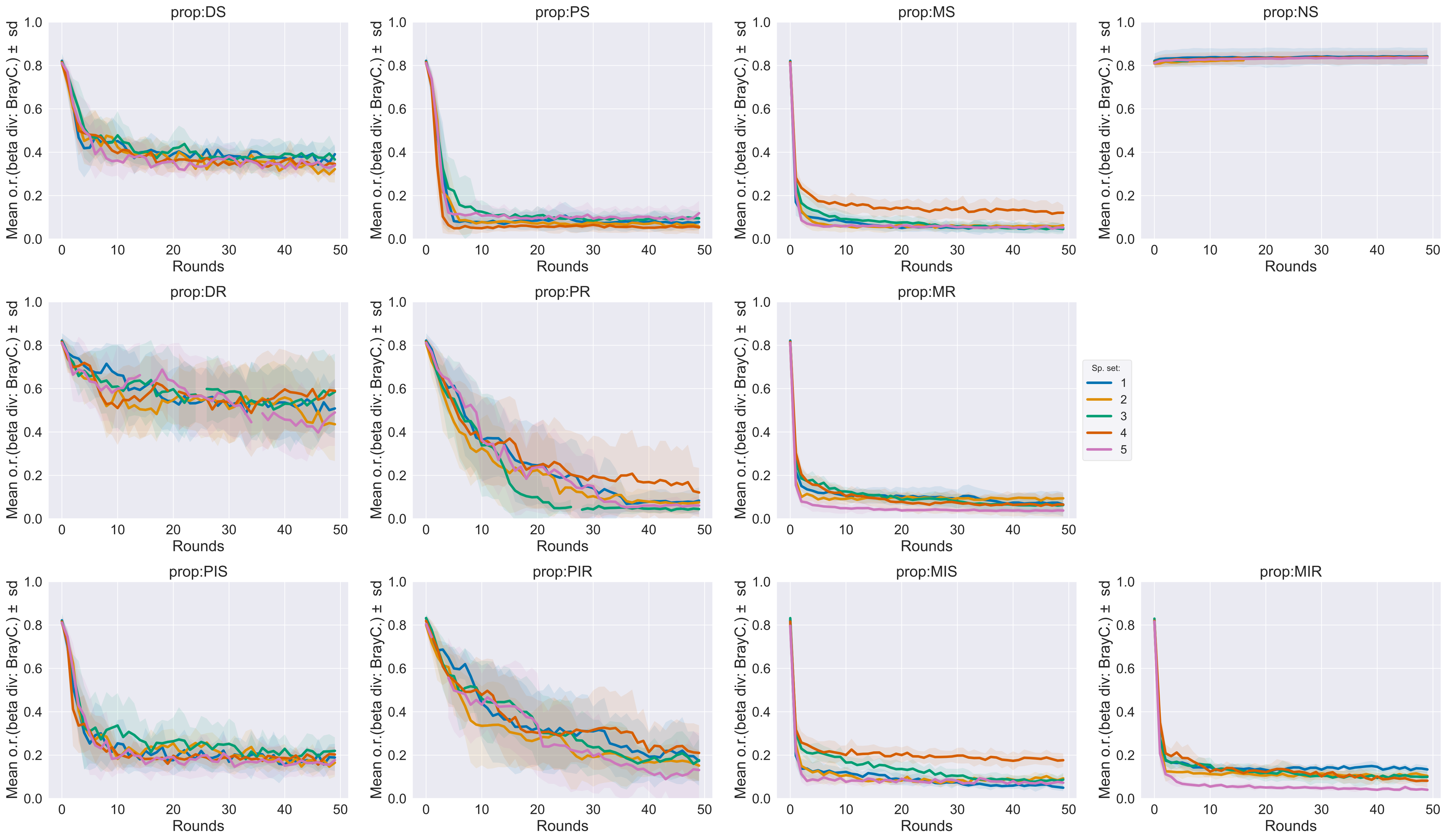

Supplement: S8 Fig — Time-series of the beta diversity corresponding to Fig 4B. Each panel shows the mean ± standard deviation over the 10 repeated runs, for each species set 1-5, for one propagation method. Data generated by the IBM. (PDF) [file pcbi.1013863.s012.pdf]

**A**Cumulative number  
of combinations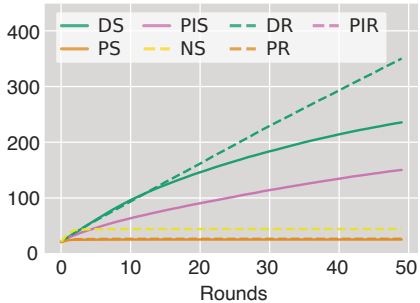**B**Average Bray-Curtis  
beta diversity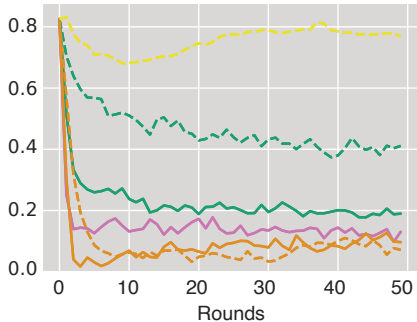

Supplement: S9 Fig — (A) Time-series of cumulative number of unique communities for each selection method, corresponding to Fig 4A but generated using the ODE model. Time-series of the beta diversity corresponding to Fig 4B, but generated using the ODE model. Each panel shows the mean ± standard deviation over the 10 repeated runs, for each species set 1-5, for one propagation method. (PDF) [file pcbi.1013863.s013.pdf]

# p-value wilcoxon rank test

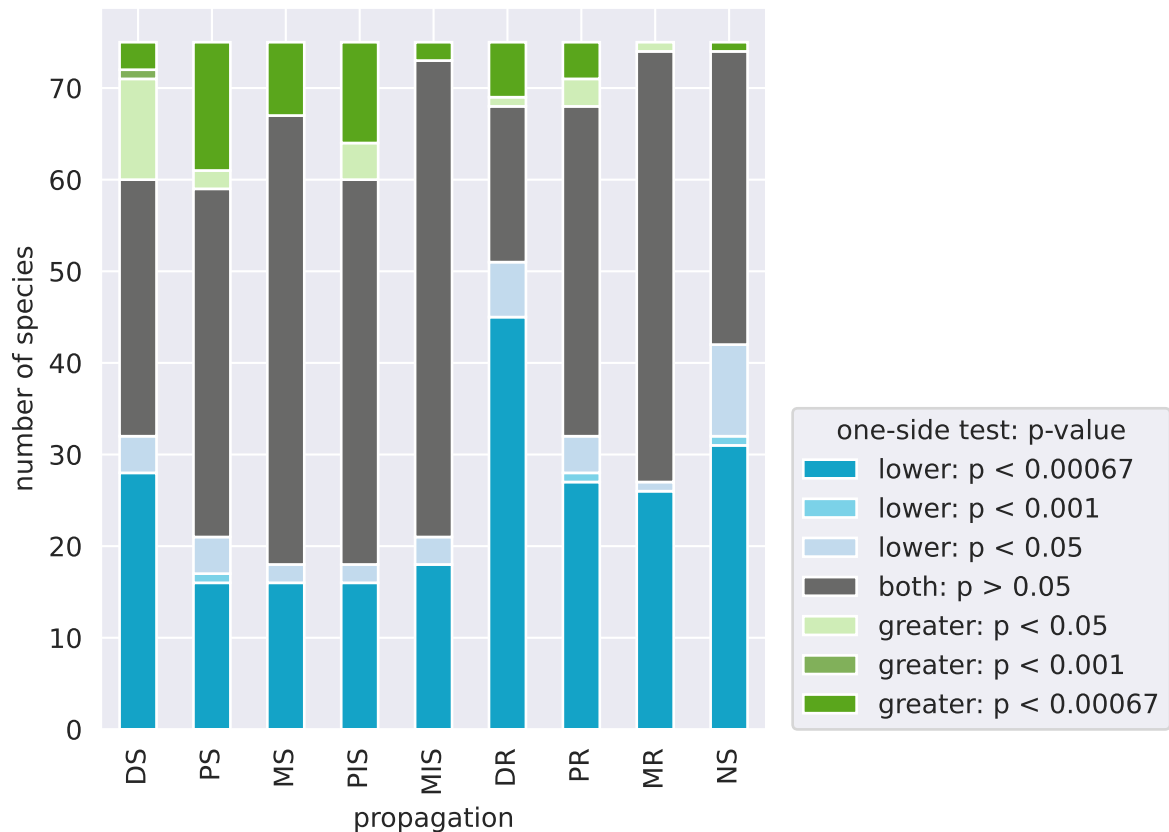

Supplement: S10 Fig — Distribution of p-values from a one-sided Wilcoxon signed-rank test of whether the total investment fl· of a species is larger/smaller in the last round where a species survived, than the investment of the ancestral species. There is one bar for each selection method, with 15 species x 5 sets of species for each bar. The alternative hypothesis is that difference in investment (ancestral-evolved) is greater (green) or less (blue) than zero. Data generated by the IBM. Data for DS is shown in Fig 5. (PDF) [file pcbi.1013863.s014.pdf]

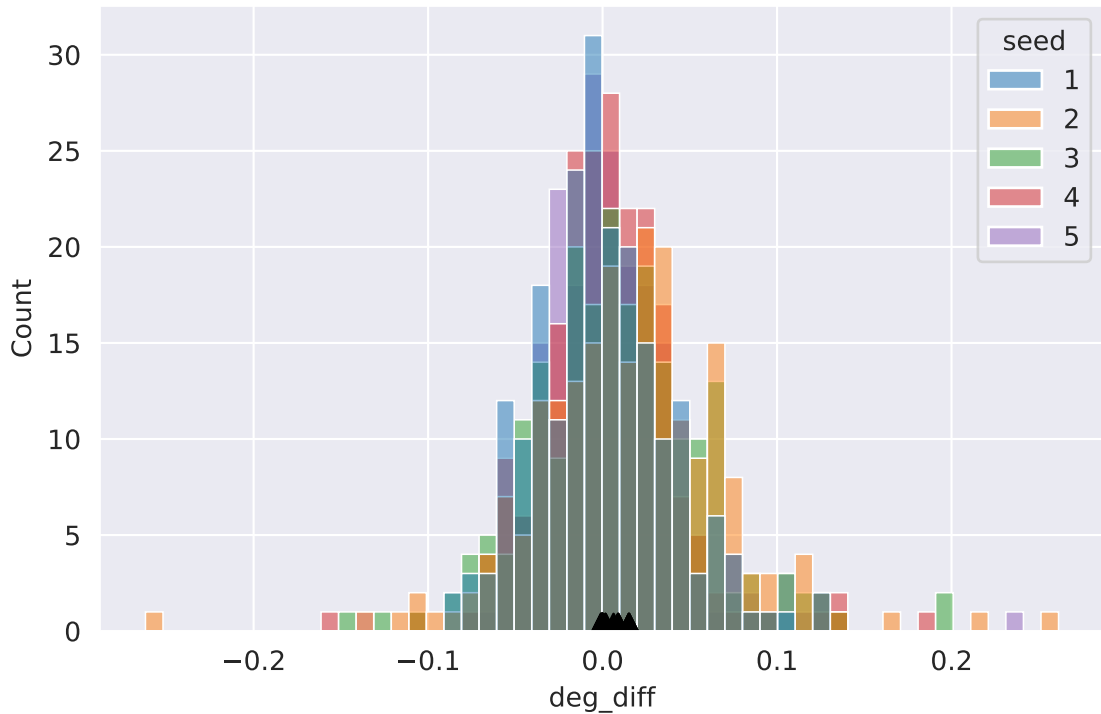

Supplement: S11 Fig — Histogram of difference in max degradation between evolved and ancestral communities. Triangles indicate the mean values for each species set. Data generated by the IBM. (PDF) [file pcbi.1013863.s015.pdf]

Difference in max. degradation between round 50 and round 0

Difference in max.  
degradation

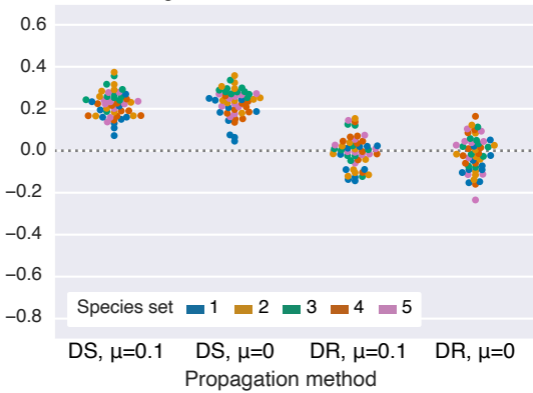

Supplement: S12 Fig — The difference in maximum degradation between round 50 and round 0 for DS and DR, corresponding to Fig 2, but varying the mutation rate from the default of μ=0.1 to μ=0. The performance of the method was not significantly affected by this change in mutation rate. (PDF) [file pcbi.1013863.s016.pdf]

Sp. set: 1

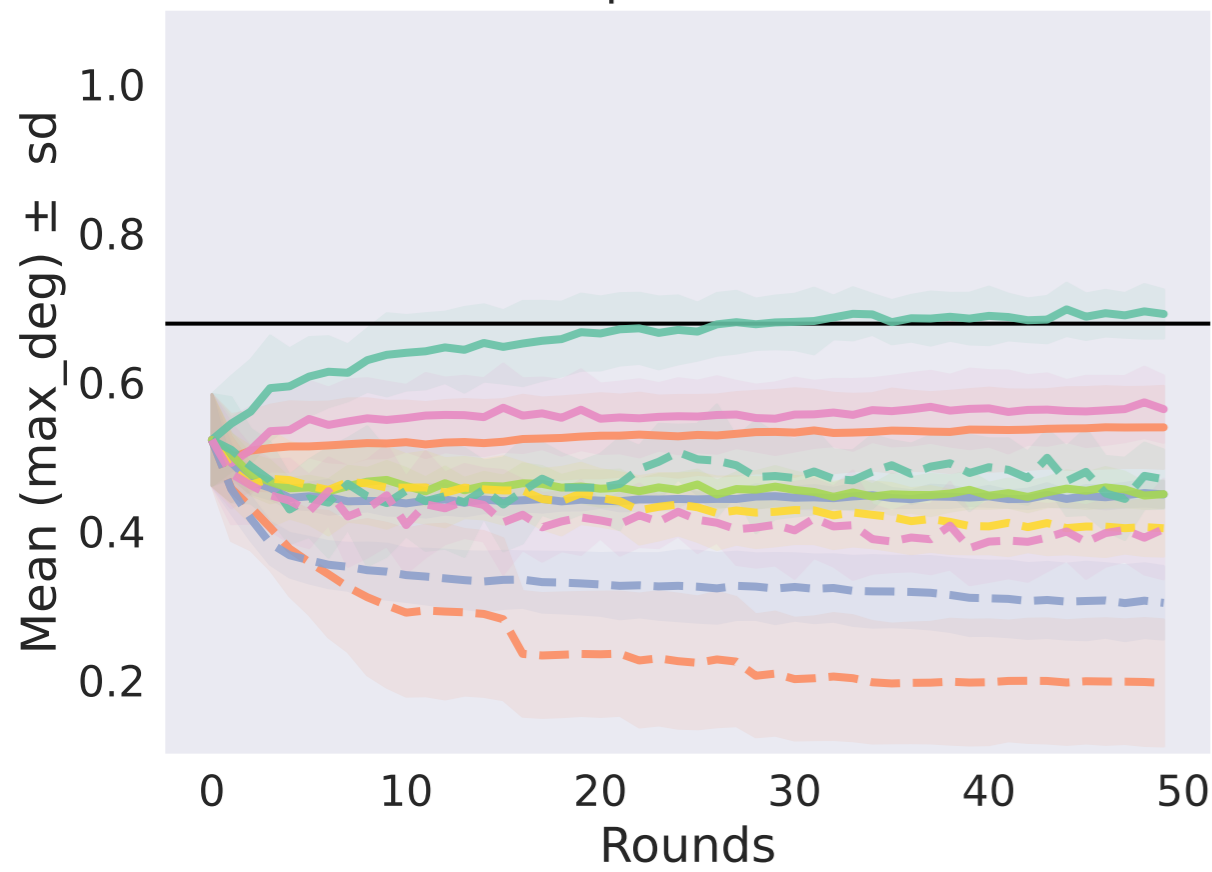

Sp. set: 2

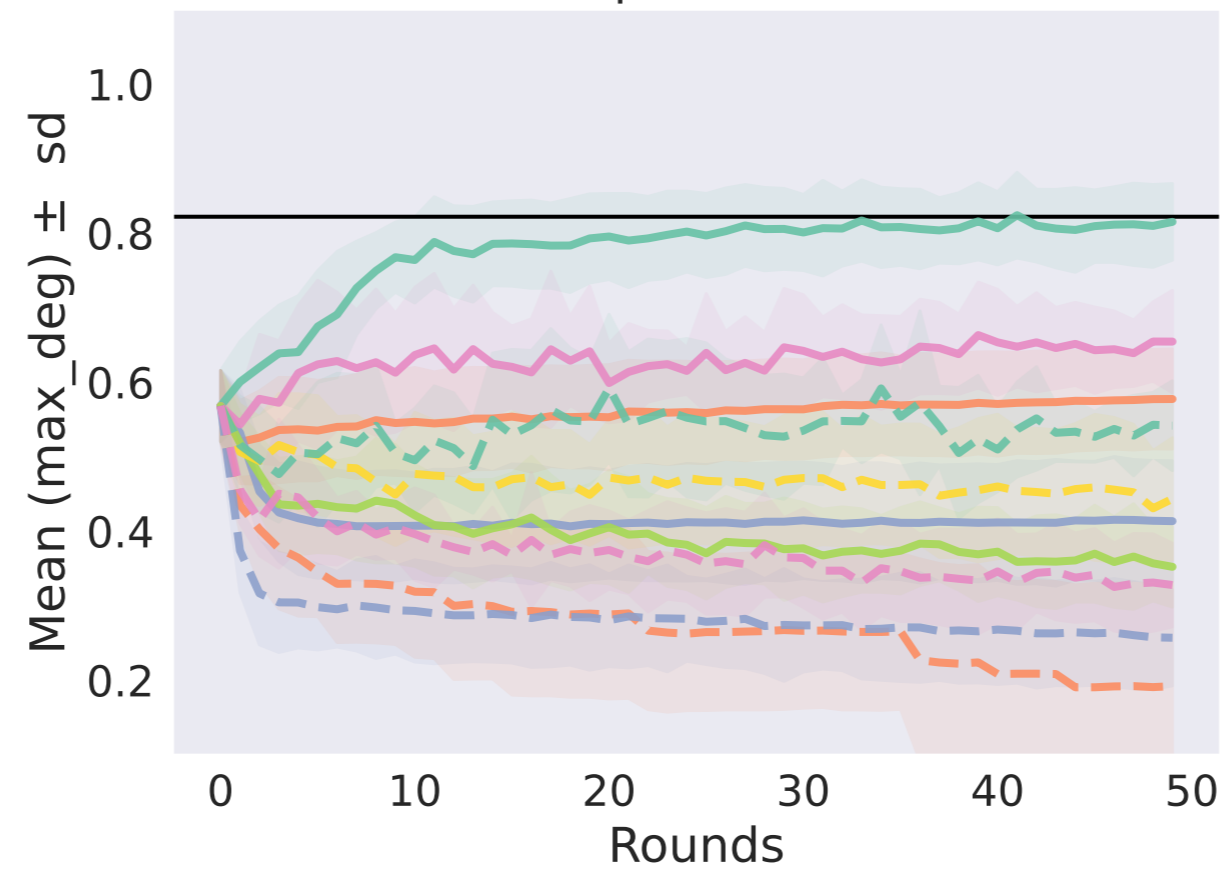

Sp. set: 3

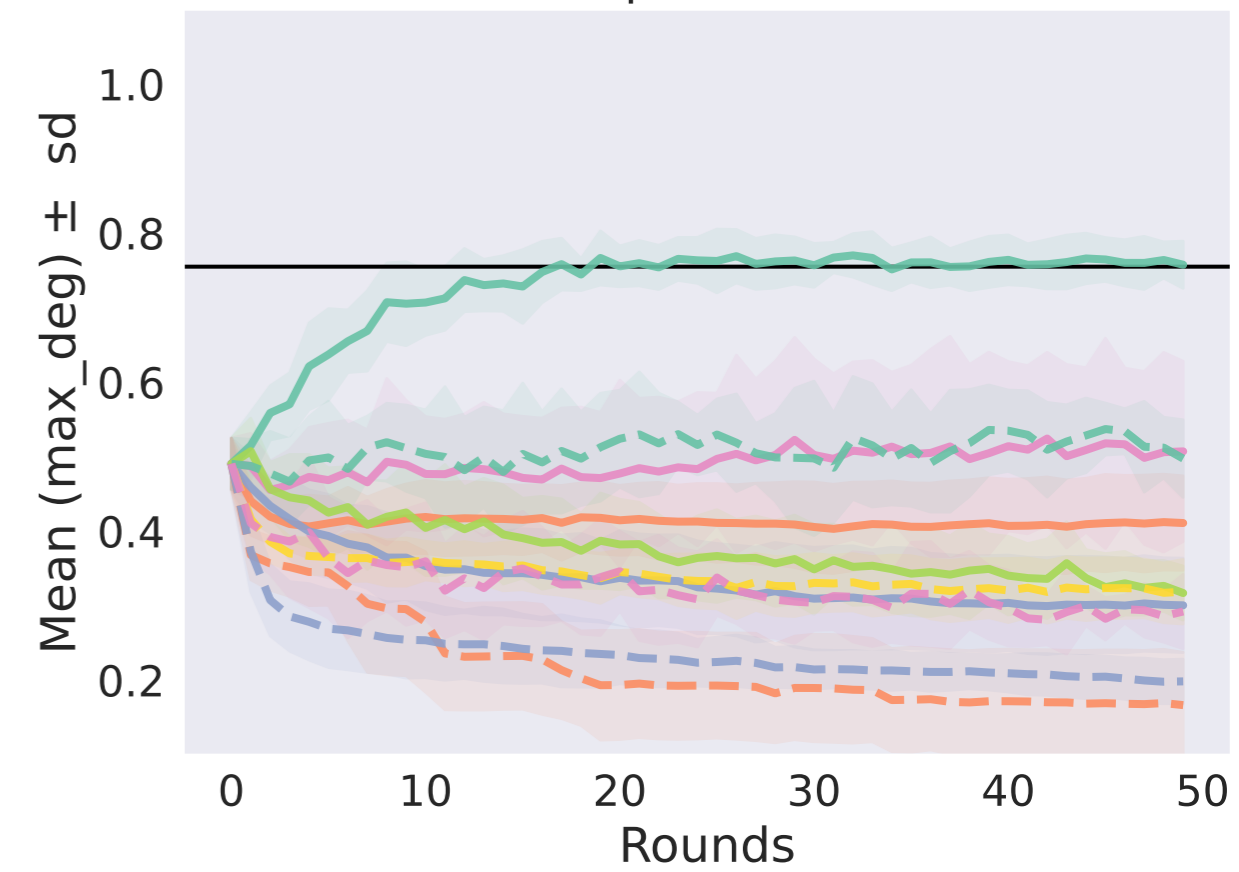

Sp. set: 4

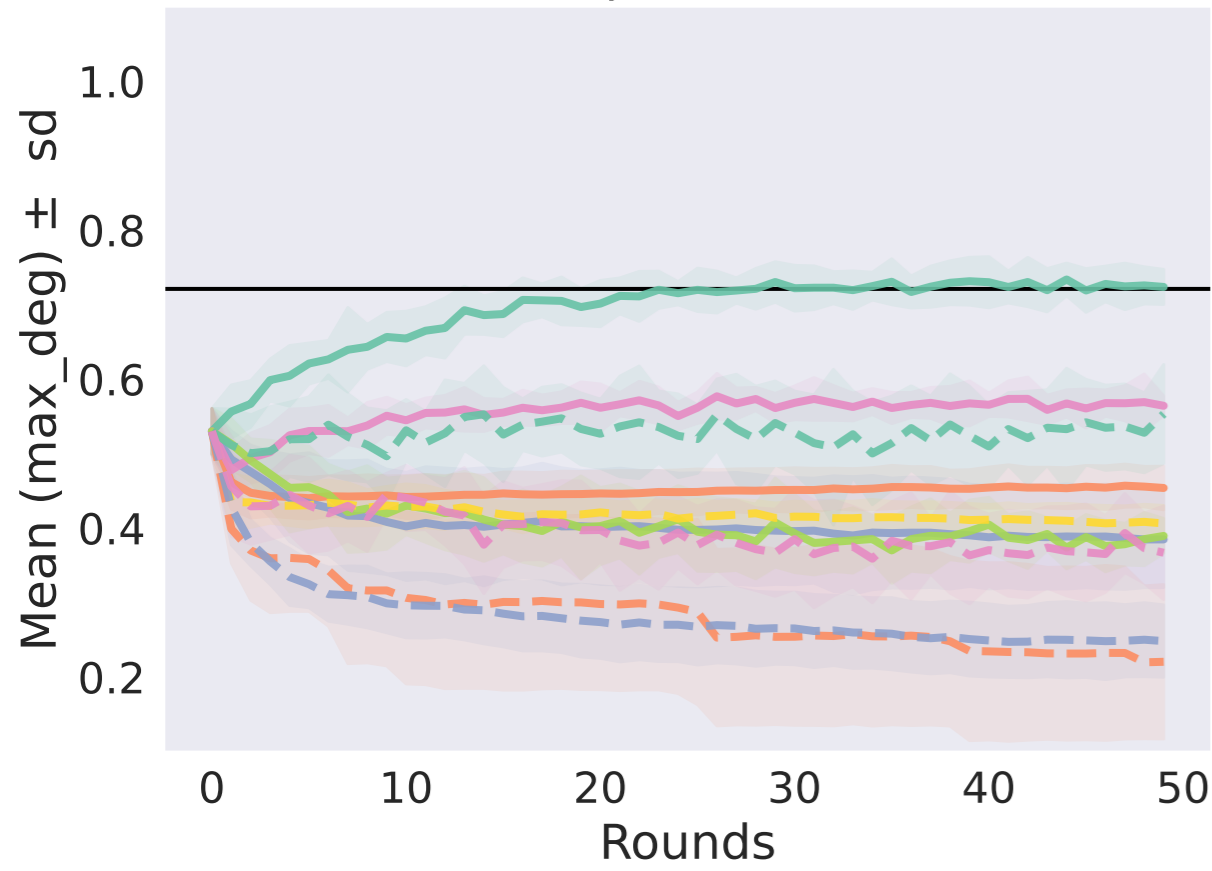

Sp. set: 5

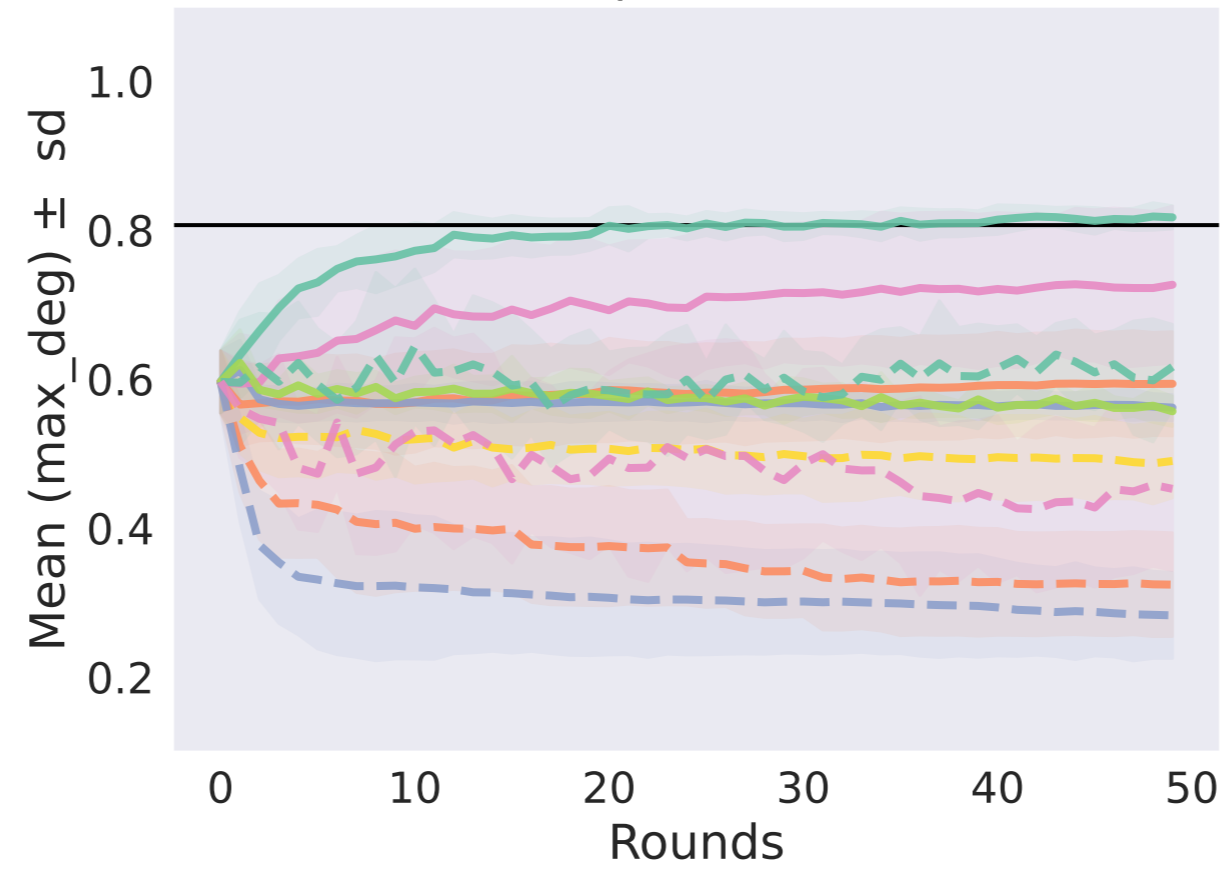

Propagation

- DS
- PS
- MS
- PIS
- MIS
- NS
- DR
- PR
- MR
- PIR

Supplement: S13 Fig — Time-series of max. degradation over 50 rounds of selection, for the different propagation methods. Each plot corresponds to one species set and shows the maximum degradation in the meta-community averaged over repeats, with the standard deviation in shades of the corresponding color. For each species set, each repeat etc, the degradation score at transfer 50 forms the swarms in Fig 2. The black line shows the degradation score of the best ancestral community out of the 32767 combinations. Data generated by the IBM. (PDF) [file pcbi.1013863.s017.pdf]

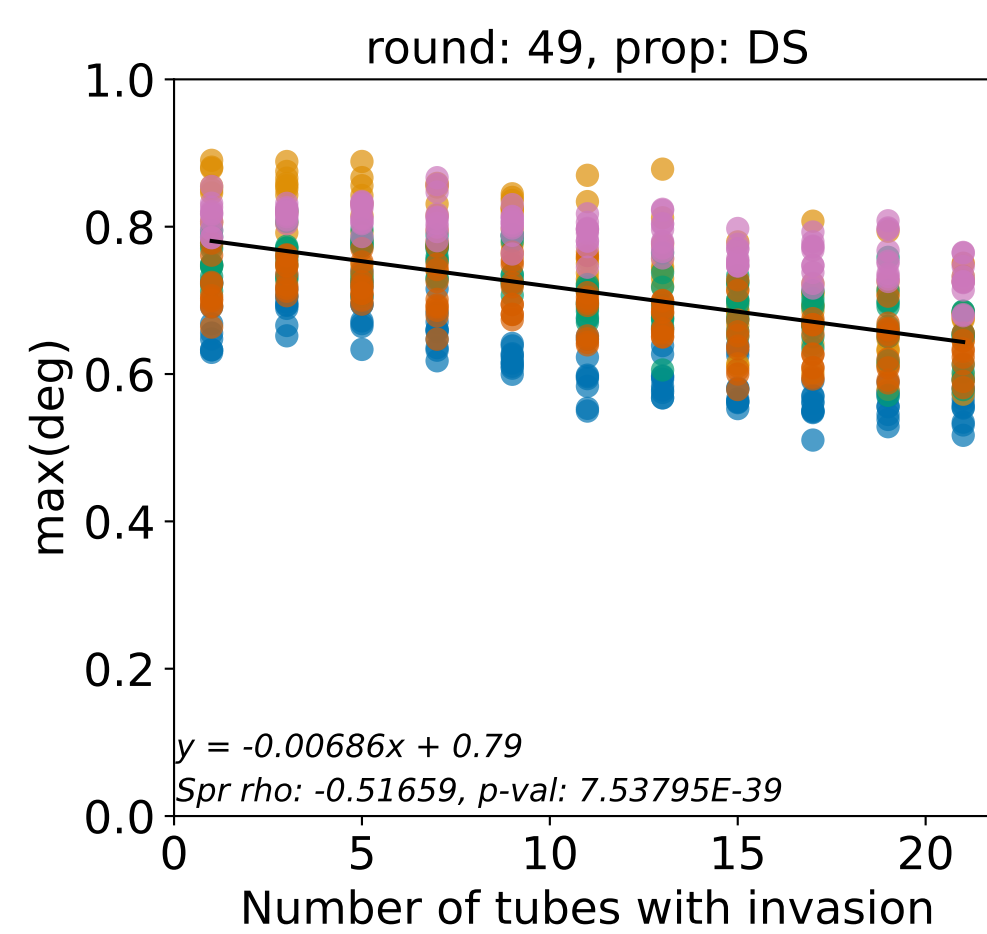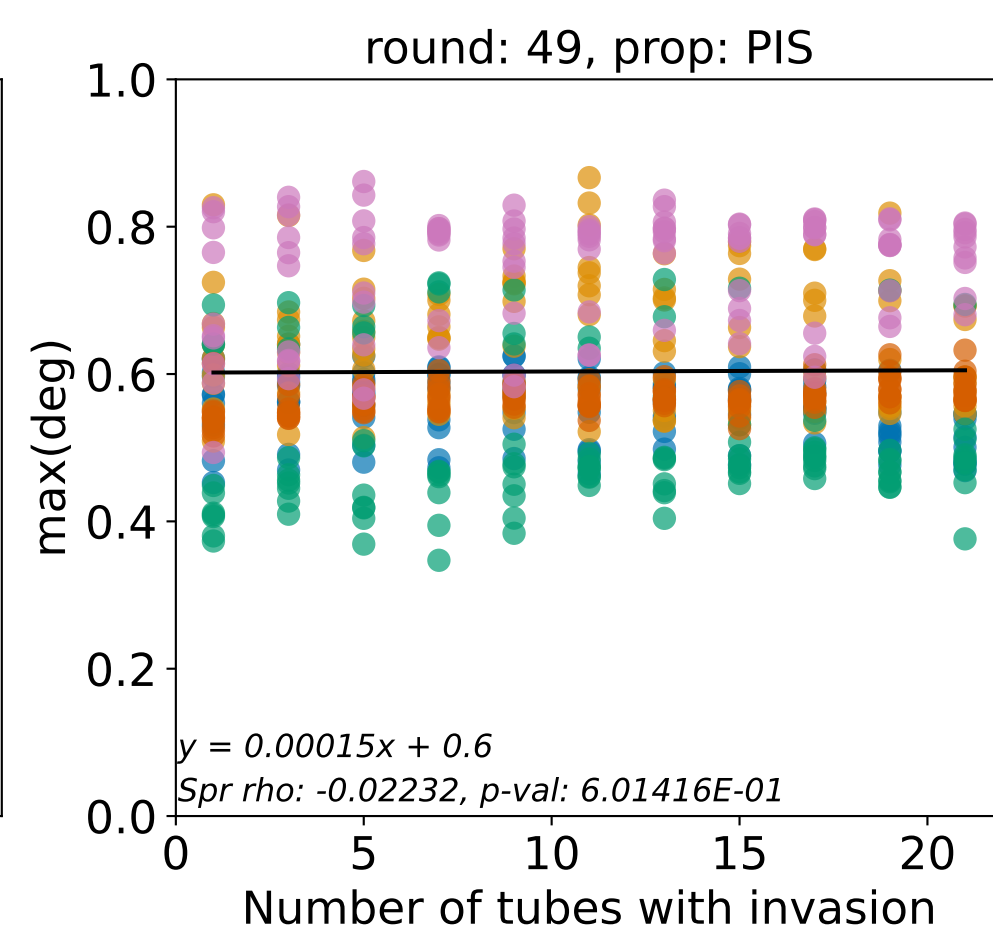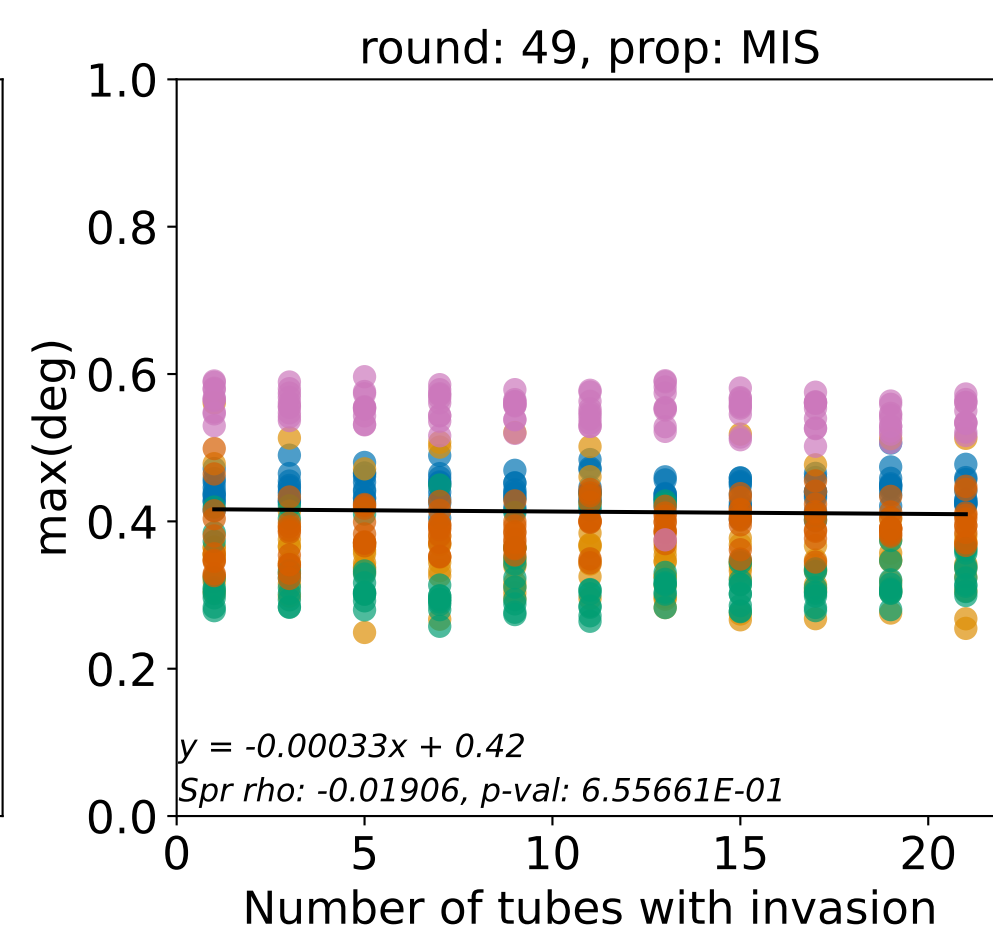

Supplement: S14 Fig — Effect on max community degradation score from changing the number of communities to receive a migrating species. Data generated by the IBM. (PDF) [file pcbi.1013863.s018.pdf]

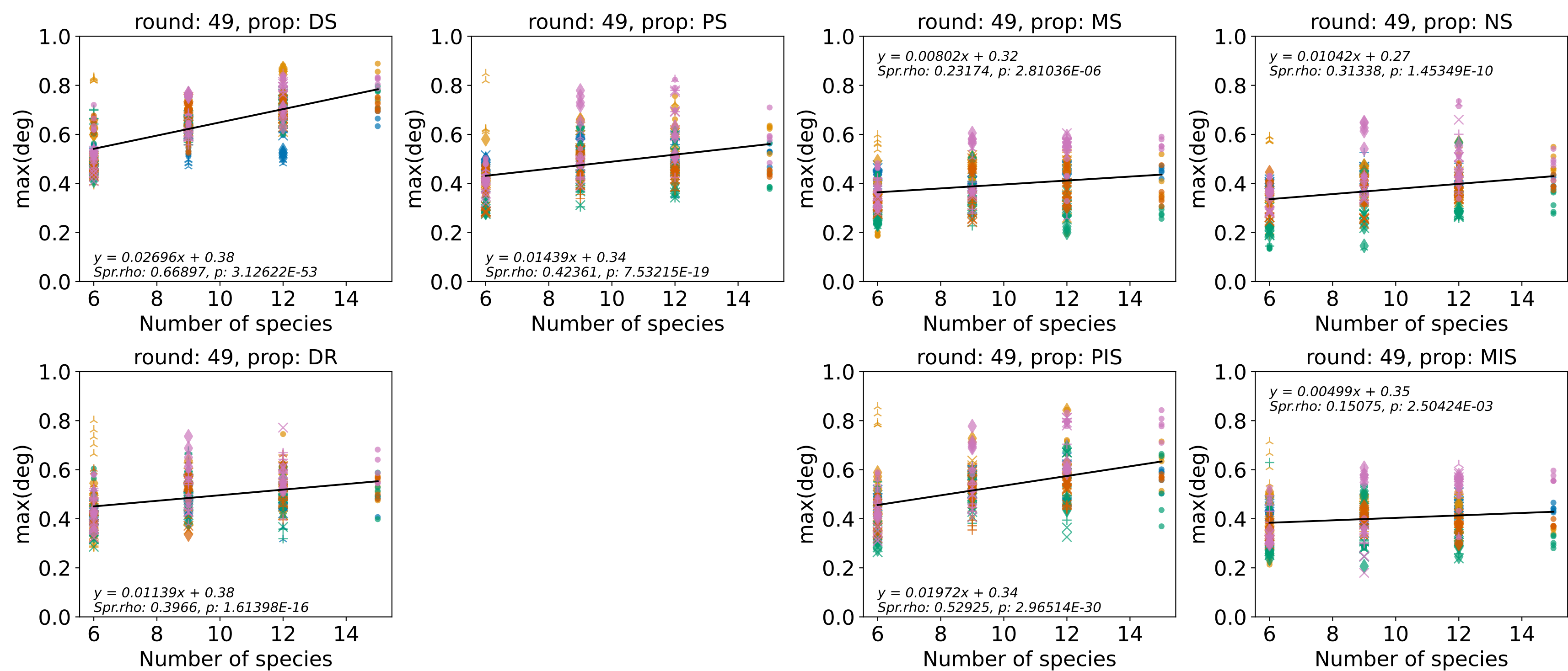

Supplement: S15 Fig — Effect on max community degradation score from changing the number of species in the ancestral community. Different marker shape indicates sub-sample (1-5) for each species group of size 6, 9, 12. For 15 species we keep the original species sets. Data generated by the IBM. (PDF) [file pcbi.1013863.s019.pdf]

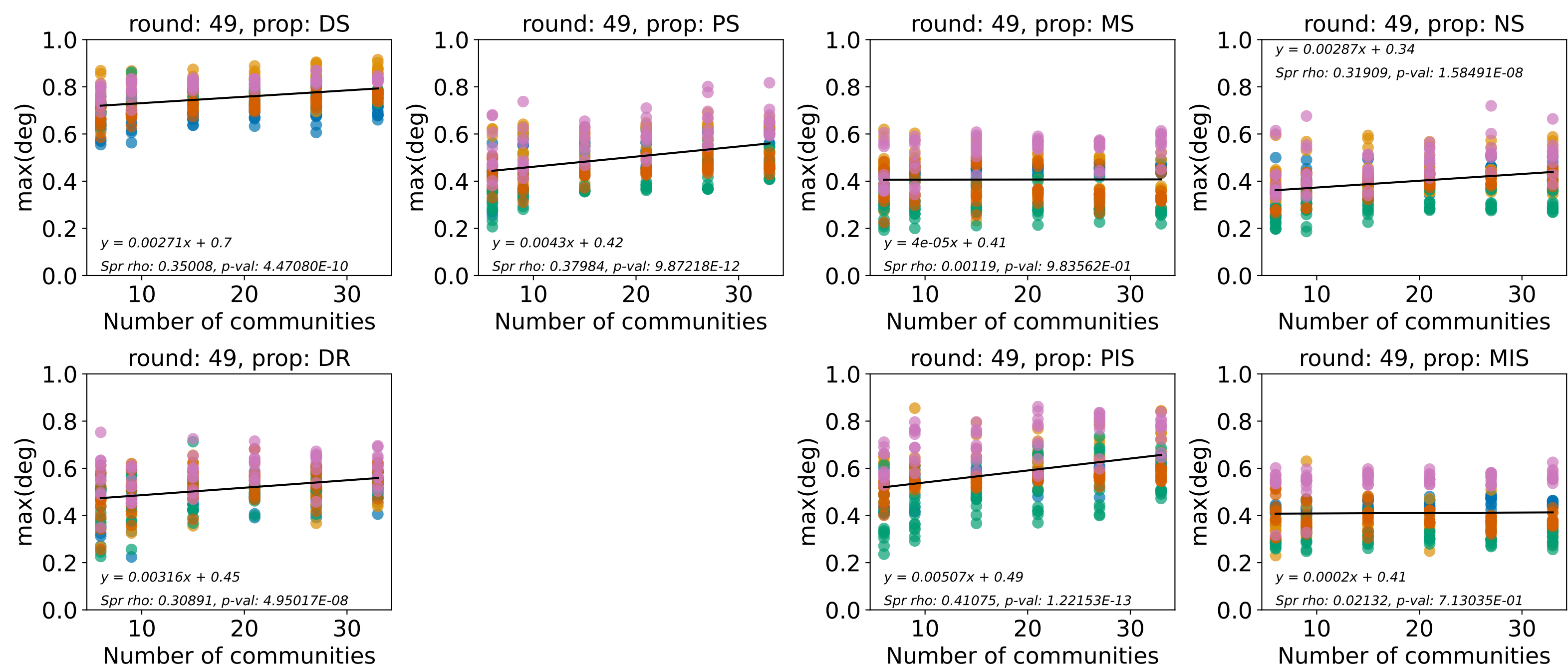

Supplement: S16 Fig — Effect on max community degradation score from changing the number of communities. Data generated by the IBM. (PDF) [file pcbi.1013863.s020.pdf]

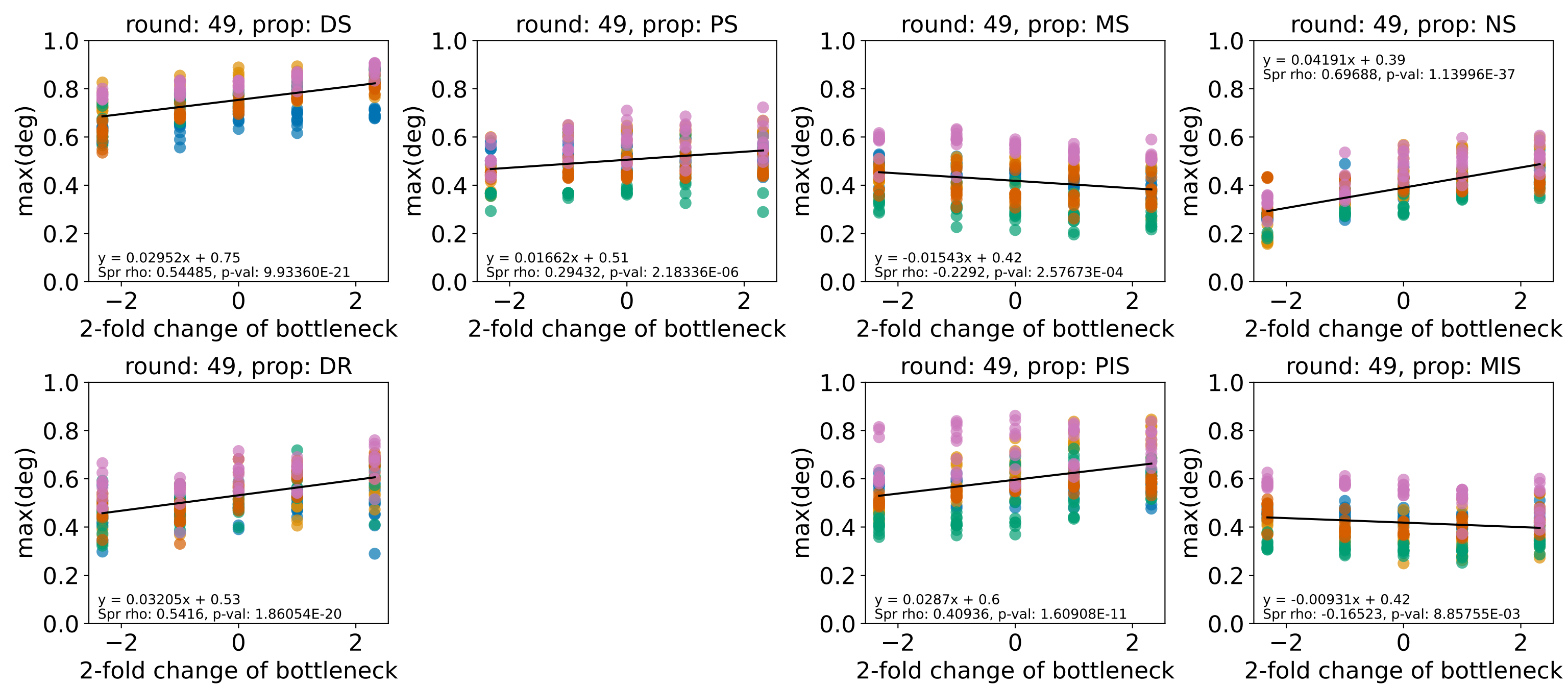

Supplement: S17 Fig — Effect on max community degradation score from scaling the dilution factor or inoculum size. Data generated by the IBM. (PDF) [file pcbi.1013863.s021.pdf]

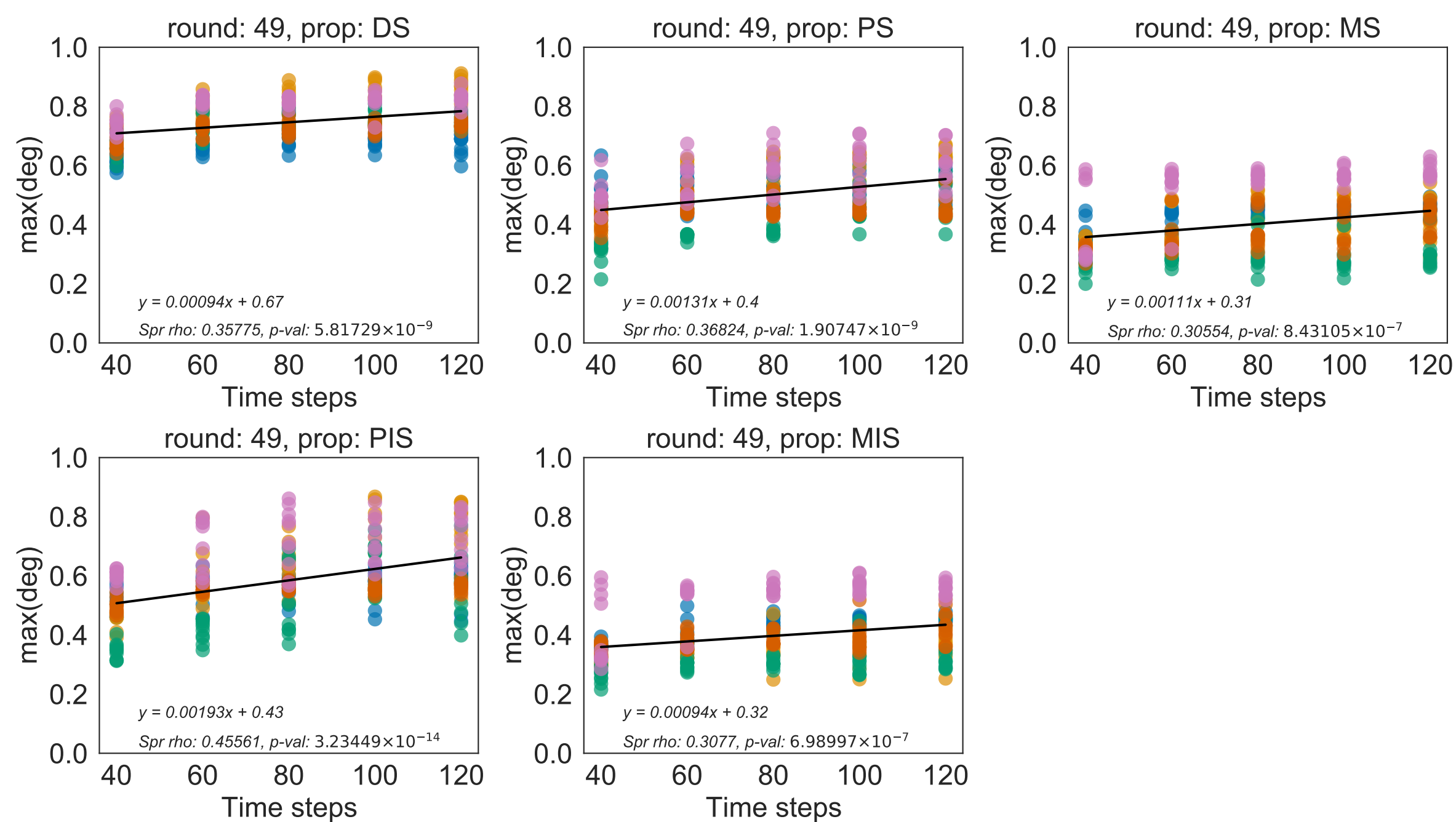

Supplement: S18 Fig — Effect on max community degradation score from changing the number of time steps in each round. Data generated by the IBM. (PDF) [file pcbi.1013863.s022.pdf]

## Comparison of Best Communities Across Species Sets

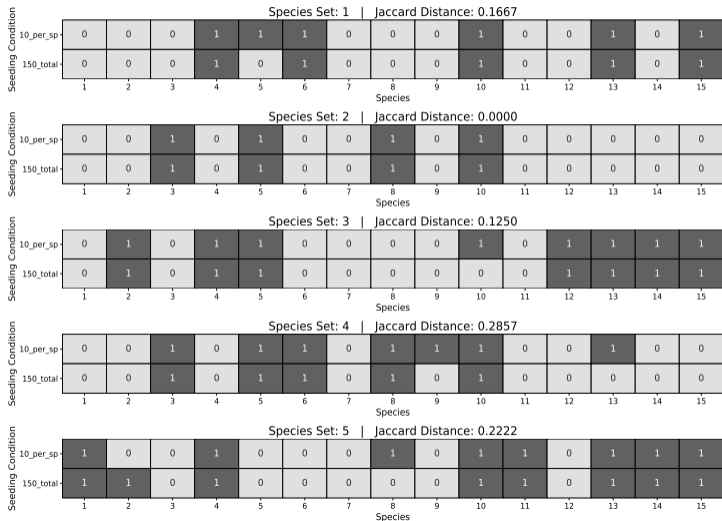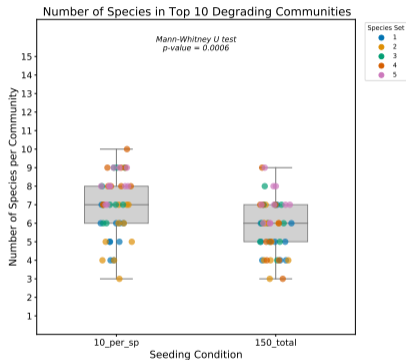

Supplement: S19 Fig — The effect of changing the inoculum size on community performance. Left: Jaccard distances between the winning communities in each species set after running 32767 species combinations with either 10 cells per species or 150 cells total. Dark grey ‘1’s and light grey ‘0’s indicate presence or absence of each of the 15 species in the winning community. This shows that the two ways of initializing our tubes resulted in very similar winning communities. Right: Number of species in each of the winning communities colored by species set in the two seeding conditions. Note that the data in the left boxplot correspond to the yellow points in Fig 3F. Data generated by the IBM. (PDF) [file pcbi.1013863.s023.pdf]
