# Supplementary material for: Novel artificial selection method improves function of simulated microbial communities
Source: PLoS Comput Biol. 2026 Jan 13;22(1):e1013863. doi: 10.1371/journal.pcbi.1013863 (PMC12829962; doi:10.1371/journal.pcbi.1013863)
Supplement: S3 Fig — Degradation scores and ranking of selected communities. (A, B) The difference in maximum degradation between round 50 and round 0 for each propagation method, corresponding to Fig 2, but generated by the ODE. (C, D) The rank of the predominant community (the most common combination of species among the 21 communities in the last round of selection, not counting sub-communities) in terms of its degradation score compared to all of the 32767 possible combinations of 1, 2, ..., 15 ancestral species, corresponding to Fig 2, but generated by the ODE. (PDF) [file pcbi.1013863.s007.pdf]

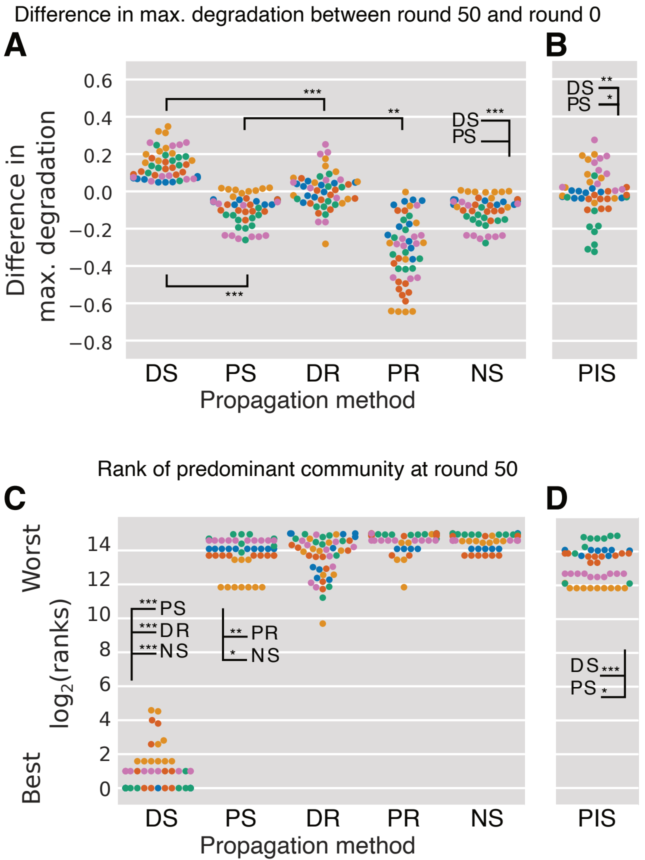
Difference in max. degradation between round 50 and round 0
A
B
0.6
0.4
0.2
0.0
−0.2
−0.4
−0.6
−0.8
Difference in
max. degradation
***
**
***
DS
PS
***
DS
PS
**
*
DS
PS
DR
PR
NS
PIS
Propagation method
Rank of predominant community at round 50
C
D
Worst
Best
log2(ranks)
14
12
10
8
6
4
2
0
*** PS
*** DR
*** NS
** PR
* NS
DS ***
PS *
DS
PS
DR
PR
NS
PIS
Propagation method
