# Supplementary material for: Novel artificial selection method improves function of simulated microbial communities
Source: PLoS Comput Biol. 2026 Jan 13;22(1):e1013863. doi: 10.1371/journal.pcbi.1013863 (PMC12829962; doi:10.1371/journal.pcbi.1013863)
Supplement: S1 Algorithm — Overall flow of the selection simulations from population growth, community dynamics and mutations to propagation by the different selection methods. (PDF) [file pcbi.1013863.s024.pdf]

Pseudo-code for implementation of models and selection methods

Input: A set of 15 species, defined by model parameters.
Input: Experimental parameters: Number of communities, time span $[t_0,\ t_{end}]$ of growth in batch. Community bottleneck $\beta = 1/3$, dilution ratio $d \in [0,\ 1]$. Initial conditions $S_i(t_0)$, $N_j(t_0)$, $T_k(t_0)$.

Assemble 21 communities by randomly drawing 4 species with replacement from the species set. Ensure that each species is present in at least one initial community;

for Each round of selection **do**

- `// Population growth, interspecies competition and invasion of mutants`
- **for** Each community **do**
  - Grow the communities for a time span $[t_0,\ t_{end}]$. (IBM implementation: S2, ODE implementation: S6);
  - Save the population sizes $S_i(t_{end})$ for each strain i in the community;
  - Save the end-state concentrations $T_k(t_{end})$ for each toxic compound k;
  - Compute the degradation score D from $T_k(t_{end})$ by (4);
- `// Propagate the communities by the chosen selection method`
- `// Required parameters: the community bottleneck` β`, dilution ratio` d
- `// Required variables: degradation scores` D `for each community`
- `// For propagule method, follow Algs. S7 and S8`
- `// For migrant pool method, IBM only, follow S9`
- `// For disassembly method, follow S10`
- `// Replenish the substrates`
- Set $N_j(t_0) = N_0$ and $T_k(t_0) = T_0$ for all j, k;

S1 Algorithm Overall flow of the selection simulations from population growth, community dynamics and mutations to propagation by the different selection methods.
