# Supplementary material for: Novel artificial selection method improves function of simulated microbial communities
Source: PLoS Comput Biol. 2026 Jan 13;22(1):e1013863. doi: 10.1371/journal.pcbi.1013863 (PMC12829962; doi:10.1371/journal.pcbi.1013863)
Supplement: S2 Algorithm — Implementation of population growth, competition and mutations in the IBM model described in the section Individual-based model. (PDF) [file pcbi.1013863.s025.pdf]

Input: A community where each microbial strain i is defined by the parameters in Tab. 1. Size of the inactive and active sub-populations p_{i0}, p_{i1} and total population size $S_i = p_{i0} + p_{i1}$. Nutrient concentrations N_j, toxic compound concentrations T_k.

Input: Mutation parameters: mutation rate μ_{mut}, trait deviation σ_m.

for Each time step **do**

  for Each community 1, ..., 21 **do**

    for Each strain i **do**

      `// Maximum uptake of nutrients of this strain, used to scale growth and degradation according to nutrient concentration`

      $max\_uptake := \sum_j (n_{ij} \text{ if } N_j > n_{ij})$;

      `// If some nutrients are depleted, re-scale` n_{ij} `to consume remaining nutrients`

      if $N_j < n_{ij}$ **then**

        $n_{ij} := 0$

      $\hat{n}_{ij} := \frac{n_{ij}}{\sum_j (n_{ij} \text{ if } N_j > n_{ij})}$;

      `// Largest number of cells that can consume the scarcest nutrient`

      $S_i^{max} := int(min(N_j / \hat{n}_{ij} \text{ if } N_j > \hat{n}_{ij}))$;

      `// Toxic compound degradation`

      if $S_i^{max} > 0$ **then**

        `// Maximal population that can degrade`

        $P_{i,tot} := \min(S_i^{max}, S_i)$;

        for Each compound T_k **do**

          if T_k *cannot be completely degraded in this time step* **then**

            $T_k := T_k - P_{i,tot} \cdot max\_uptake \cdot f_{ik}$;

            for Each nutrient N_j **do**

              $N_j := N_j - \hat{n}_{ij} \cdot P_{i,tot} \cdot f_{ik}$;

          else

            Degrade remaining toxic compounds and consume corresponding nutrients

      `// Cell division step 1: Costly activation` S3

  `// Cell division step 2: Replication` S4

  `// Cell death:` S5

S2 Algorithm Implementation of population growth, competition and mutations in the IBM model.
