# Supplementary material for: Novel artificial selection method improves function of simulated microbial communities
Source: PLoS Comput Biol. 2026 Jan 13;22(1):e1013863. doi: 10.1371/journal.pcbi.1013863 (PMC12829962; doi:10.1371/journal.pcbi.1013863)
Supplement: S3 Algorithm — Activation of cells, the first step of cell division in the IBM described in S2 Algorithm. (PDF) [file pcbi.1013863.s026.pdf]

Input: Communities where each strain i is defined by parameters in Tab. 1. Inactive and active sub-populations p_{i0}, p_{i1}. The maximal population S_i^{max} that can afford to consume nutrients, based on their current availability. Re-scaled nutrient consumption rates $\hat{n}_{ij}$. Current nutrient concentrations N_j.

Input: Parameters: Initial nutrient concentration N_0.

`// Cell activation`

if $S_i^{max} > 0$ **then**

    `// Already activated cells consume nutrients`

    if $S_i^{max} \geq p_{i1}$ **then**

        $N_j := N_j - \hat{n}_{ij} \cdot p_{i1} \cdot (1 - \sum_k f_{ik})$;

        $S_i^{max} := S_i^{max} - p_{i1}$;

    else

        Deactivate cells that cannot afford to stay activated, consume nutrients for remaining activated cells, set $S_i^{max} := 0$

    `// Newly activated cells`

    $cells\_activate :=$ Poisson$(a_i \cdot p_{i0} \cdot max_uptake \cdot (1 - \sum_k f_{ik}) \cdot \sum_j (\hat{n}_{ij} \cdot N_j / N_0))$;

    if $cells\_activate > p_{i0}$ **then**

        $cells\_activate := p_{i0}$

    if $cells\_activate > S_i^{max}$ **then**

        $cells\_activate := S_i^{max}$

    $p_{i0} := p_{i0} - cells\_activate$;

    $p_{i1} := p_{i1} + cells\_activate$;

    for Each nutrient N_j **do**

        $N_j := N_j - \hat{n}_{ij} \cdot cells\_activate \cdot (1 - \sum_k f_{ik})$;

else

    Deactivate all activated cells

return *Populations* p_{i0}, p_{i1} *for strain* i*, current nutrient concentrations* N_j

S3 Algorithm Activation of cells, first step of cell division in the IBM.
