# Supplementary material for: Novel artificial selection method improves function of simulated microbial communities
Source: PLoS Comput Biol. 2026 Jan 13;22(1):e1013863. doi: 10.1371/journal.pcbi.1013863 (PMC12829962; doi:10.1371/journal.pcbi.1013863)
Supplement: S4 Algorithm — Replication and mutation, second step of cell division of the IBM described in S2 Algorithm. (PDF) [file pcbi.1013863.s027.pdf]

Input: Communities where each strain i is defined by parameters in Tab. 1.
Inactive and active sub-populations p_{i0}, p_{i1}.

```
for Each community do
    for Each strain i in the community do
        // Calculate the number of new cells appearing due to division
        new_cells := Poisson(p_i1 · r_i · (1 − Σ_k f_ik));
        if new_cells > p_i1 then
            new_cells := p_i1
        // Calculate how many new_cells will carry mutations
        mutants := Poisson(new_cells · μ_mut);
        if mutants > new_cells then
            mutants := new_cells
        p_i1 := p_i1 − new_cells;
        p_i0 := p_i0 + new_cells · 2 − mutants;
        // Mutation
        for Each new mutant do
            Add a new strain i to the community, with the model parameters of
              the ancestor and set p_i0 := 1 and p_i1 := 0;
            Decide which f_ik to mutate by drawing from Bernoulli(1/N_tox) for each
              f_ik; ensure that at least one f_ik mutates;
            for Each successful draw do
                Multiply the chosen f_ik by a factor x ~ lognormal(0.0, σ_m);
                Re-scale so Σ_k f_ik ≤ 1, if needed;
return Populations p_i0, p_i1 for each strain i, including the new ones resulting
  from mutation.
```

S4 Algorithm Replication and mutation, second step of cell division of the IBM described in S2.
