# Supplementary material for: Novel artificial selection method improves function of simulated microbial communities
Source: PLoS Comput Biol. 2026 Jan 13;22(1):e1013863. doi: 10.1371/journal.pcbi.1013863 (PMC12829962; doi:10.1371/journal.pcbi.1013863)
Supplement: S5 Algorithm — Cell death of the IBM described in S2 Algorithm. (PDF) [file pcbi.1013863.s028.pdf]

Input: Communities where each strain i is defined by parameters in Tab. 1. Inactive and active sub-populations p_{i0}, p_{i1}. Current concentrations T_k of toxic compounds. Death rates m_{ik} and K constant for the Hill function.

for Each community **do**

  for Each strain i, looping over the community in reverse order **do**

    $p_{i0} := p_{i0} -$ Poisson$\left(p_{i0} \cdot \sum_k \left(m_{ik} \cdot \frac{T_k^2}{T_k^2+K^2}\right)\right)$; ensure that $p_{i0} \geq 0$;

    $p_{i1} := p_{i1} -$ Poisson$\left(p_{i1} \cdot \sum_k \left(m_{ik} \cdot \frac{T_k^2}{T_k^2+K^2}\right)\right)$; ensure that $p_{i1} \geq 0$;

    if $p_{i0} + p_{i1} = 0$ **then**

      remove strain i from the community

  Randomly shuffle strains in community;

return *Populations* p_{i0}, p_{i1} *for each strain* i*.*

S5 Algorithm Cell death of the IBM described in S2.
