# Supplementary material for: Novel artificial selection method improves function of simulated microbial communities
Source: PLoS Comput Biol. 2026 Jan 13;22(1):e1013863. doi: 10.1371/journal.pcbi.1013863 (PMC12829962; doi:10.1371/journal.pcbi.1013863)
Supplement: S6 Algorithm — Implementation of population growth, competition and mutations in the ODE model described in the section Population-level model. To solve the equations, we use dopri5 from the SciPy library [43,46]. (PDF) [file pcbi.1013863.s029.pdf]

Input: Strains with model parameters from S4 for each population i.
Input: Mutation parameters: rate μ_{mut}, trait deviation σ_m.
Input: Experimental parameters: Number of toxic compounds N_{tox}, initial concentrations N_0, T_0 of nutrients and toxic compounds, initial population size S_0. Time span for growth $[t_0,\ t_{end}]$.

for Each community **do**
  `// Growth and competition within one round`
  Solve the equations (11)–(13) for a time span $[t_0,\ t_{end}]$;
  Save the end states $S_i(t_{end})$, $N_j(t_{end})$, $T_k(t_{end})$;
  `// Mutations, ODE model`
  for Each strain i, with probability μ_{mut} **do**
    Copy the species parameters to an empty place in the list of populations;
    Choose a f_{ik} at random by drawing from Bernoulli$(1/N_{tox})$ for each $k = 1, \ldots, 10$;
    For each chosen f_{ik}, multiply by a factor $x_k \sim \text{lognormal}(0.0, \sigma_m)$;
    Set the inoculum size to S_0;

return $S_i(t_{end}),\ N_j(t_{end}),\ T_k(t_{end}),\ f_{ik}$

S6 Algorithm Implementation of population growth, competition and mutations in the ODE model described in the section *Population-level model*. To solve the equations, we use *dopri5* from the SciPy library [43, 46].
