# Supplementary material for: Novel artificial selection method improves function of simulated microbial communities
Source: PLoS Comput Biol. 2026 Jan 13;22(1):e1013863. doi: 10.1371/journal.pcbi.1013863 (PMC12829962; doi:10.1371/journal.pcbi.1013863)
Supplement: S7 Algorithm — Implementation of the propagule selection method for the ODE model. (PDF) [file pcbi.1013863.s030.pdf]

Input: Communities with populations S_i and degradation scores D. End states $T_k(t_{end})$.
Input: Experimental parameters: selection bottleneck $\beta = 1/3$, dilution ratio d.
Rank the communities by degradation D;
Select the top $N_\beta = 7$ of communities with the highest ranks;
`// Re-populate the new set of tubes`
Allocate $1/\beta$ new tubes for each selected community;
for Each selected community $1, 2, \ldots, 7$ **do**
    for Each population S_i in the selected community **do**
        `// Dilute the population`
        Dilute $S_i(t_0) := d \cdot S_i(t_{end})$;
        if $S_i(t_0) < 1.0$ **then**
            `// The population is extinct`
            Set $S_i(t_0) := 0.0$;
            Remove all species parameters from the community;
        Copy model parameters and population sizes $S_i(t_0)$ of each strain in the parent communities to each of the $1/\beta$ offspring communities;

S7 Algorithm Implementation of the propagule selection method for the ODE model.
