# Supplementary material for: Novel artificial selection method improves function of simulated microbial communities
Source: PLoS Comput Biol. 2026 Jan 13;22(1):e1013863. doi: 10.1371/journal.pcbi.1013863 (PMC12829962; doi:10.1371/journal.pcbi.1013863)
Supplement: S8 Algorithm — Implementation of the propagule selection method for the IBM. (PDF) [file pcbi.1013863.s031.pdf]

**Input:** Communities with degradation scores $D$ and strains $S_i$ with total population $S_i = p_{i0} + p_{i1}$. End-state concentration of toxic compounds $T_k(t_{\text{end}})$.
**Input:** Experimental parameters: selection bottleneck $\beta = 1/3$, dilution ratio $d$.
Rank the communities by $D$;
Select the top $N_\beta = 7$ communities with the highest ranks;
`// Re-populate the new set of tubes`
Allocate $1/\beta$ new tubes for each selected community;
**for** each selected community $1, 2, \ldots, 7$ **do**
  **for** each strain $i$ in the community **do**
    `// Deactivate cells`
    $p_{i0}(t_{\text{end}}) = S_i(t_{\text{end}})$;
    $p_{i1}(t_{\text{end}}) = 0$;
    `// Dilute the population; new cells will be inactivated`
    $p_{i0}(t_0) := \text{Poisson}(d \cdot S_i(t_{\text{end}}))$;
    **if** $p_{i0}(t_0) > S_i(t_{end})$ **then**
      $p_{i0}(t_0) = S_i(t_{\text{end}})$
    **if** $p_{i0}(t_0) > 0$ **then**
      Append strain i with population $p_{i0}(t_0)$ to the new tube
    ;
    `// Delete selected cells to not choose them again`
    $S_i(t_{\text{end}}) = S_i(t_{\text{end}}) - p_{i0}(t_0)$;

**S8 Algorithm** Implementation of the propagule selection method for the IBM.
