# Supplementary material for: Novel artificial selection method improves function of simulated microbial communities
Source: PLoS Comput Biol. 2026 Jan 13;22(1):e1013863. doi: 10.1371/journal.pcbi.1013863 (PMC12829962; doi:10.1371/journal.pcbi.1013863)
Supplement: S9 Algorithm — Implementation of the migrant pool selection method for the IBM. (PDF) [file pcbi.1013863.s032.pdf]

Input: Communities with degradation scores D and strains S_i with total population $S_i = p_{i0} + p_{i1}$. End-state concentration of toxic compounds $T_k(t_{end})$.

Input: Experimental parameters: selection bottleneck $\beta = 1/3$, dilution ratio d.

Rank the communities by D;

Select the top $N_\beta = 7$ communities with the highest ranks, and pool their populations;

`// Re-populate the new set of tubes`

Allocate 21 new tubes;

for Each offspring community $1,\ 2,\ \ldots,\ 21$ **do**

- **for** Each strain i in the pool **do**
  - `// Deactivate cells`
  - $p_{i0}(t_{end}) = S_i(t_{end})$;
  - $p_{i1}(t_{end}) = 0$;
  - `// Dilute the population; new cells will be inactivated`
  - Draw $p_{i0}(t_0)$ from Poisson$\left(\frac{d}{N_\beta} \cdot S_i(t_{end})\right)$;
  - **if** $p_{i0}(t_0) > S_i(t_{end})$ **then**
    - $p_{i0}(t_0) = S_i(t_{end})$
  - **if** $p_{i0}(t_0) > 0$ **then**
    - Append strain i with population $p_{i0}(t_0)$ to the new tube
  - ;
  - `// Delete selected cells to not choose them again`
  - $S_i(t_{end}) = S_i(t_{end}) - p_{i0}(t_0)$;

S9 Algorithm Implementation of the migrant pool selection method for the IBM
