# Supplementary material for: Novel artificial selection method improves function of simulated microbial communities
Source: PLoS Comput Biol. 2026 Jan 13;22(1):e1013863. doi: 10.1371/journal.pcbi.1013863 (PMC12829962; doi:10.1371/journal.pcbi.1013863)
Supplement: S10 Algorithm — Implementation of the disassembly method. (PDF) [file pcbi.1013863.s033.pdf]

Input: Communities with populations S_i (in the IBM $S_i = p_{i0} + p_{i1}$) with model parameters from (Tab. 1, Tab. S4) and degradation scores D.
Input: Experimental parameters: selection bottleneck $\beta = 1/3$, initial population size S_0 and number of new communities to emigrate species from $N_{emi} = 5$, and immigrate species to $N_{immi} = 5$.

`// Rank the communities`
Rank the communities by the degradation score D;
`// Update the fossil record with the top communities in this round`
for Each selected community 1, 2, ..., 7 **do**
    for Each species l in the selected community **do**
        if The record of species l is not yet updated in this round of selection **then**
            Add all strains i of species l to the fossil record, including the corresponding parameters and population sizes;

`// Propose new communities in proportion to their degradation scores and survival`
Follow S11;
`// Emigration`
Draw $N_{emi} = 5$ communities with uniform probability;
for Each chosen community 1, ..., N_{emi} **do**
    `// Find emigrating species`
    $Number\_of\_emigrants = 1 + \text{Poisson}(0.5)$;
    Verify that at least one species will remain;
    for Each emigrant **do**
        Choose the emigrant at random, with priority for species that occur in more than one community;
        Remove all strains of this species from the community;

`// Immigration`
Draw $N_{immi} = 5$ communities with uniform probability;
for Each chosen community 1, ..., N_{immi} **do**
    `// Find immigrating species`
    $Number\_of\_immigrants = 1 + \text{Poisson}(0.5)$;
    for Each immigrant **do**
        if *There are species that do not feature in any community* **then**
            Choose one of them at random
        else
            Choose a species that is not already in the community with uniform probability
        ;
        Take all strains of the species from the species record, add them to the offspring community;
        Set the population size to S_0 (approximately S_0 in the IBM, see S11), in proportion to strain relative abundance;

S10 Algorithm Implementation of the disassembly method.
