# Supplementary material for: Novel artificial selection method improves function of simulated microbial communities
Source: PLoS Comput Biol. 2026 Jan 13;22(1):e1013863. doi: 10.1371/journal.pcbi.1013863 (PMC12829962; doi:10.1371/journal.pcbi.1013863)
Supplement: S11 Algorithm — Method to propose new communities based on their degradation scores from the previous round. Called by S10 Algorithm. (PDF) [file pcbi.1013863.s034.pdf]

Input: The subset of selected communities, their degradation scores D. Population sizes S_i of all strains (in the IBM, $S_i = p_{i0} + p_{i1}$).

Input: Parameters: Number of species N_{spc} that were inoculated in the different communities. Inoculum size per species S_0.

`// Scale degradation scores by species extinctions`

for Each selected community $1,\ 2,\ \ldots,\ 7$ **do**

- `// Count the number of surviving species in this community`
- Set the extinction counter $E = 0$;
- **for** Each species l in the community **do**
  - **if** $S_i = 0$ *for all strains of species* l **then**
    - Set $E := E + 1$;
    - `// Re-introduce species` l `from the record`
    - Take all strains of species l from the most recent record;
- `// Scale the degradation score` D `by the fraction of surviving species`
- Set $\hat{D} := D \cdot (N_{spc} - E)/N_{spc}$;

`// Calculate a probability distribution based on degradation scores`

Set $p_n := \hat{D}_n / \sum_m \hat{D}_m$ for each community n;

`// Propose new communities randomly in proportion to` p_n

for Each offspring community **do**

- Choose a parental community at random, by the probability distribution p_n;
- **for** Each species l in the parental community **do**
  - Copy the growth parameters and f_{ik} of all strains i to the offspring community;
  - `// ODE: Set the population size of each species to` S_0 `in total, in proportion to the relative abundance of the strains.`
  - `// IBM: Sample approximately` S_0 `cells with replacement as follows:`
  - **for** Each strain i (with population S_i) of species l (with population S_l) **do**
    - `// Deactivate cells`
    - $p_{i0}(t_{end}) = S_i(t_{end})$;
    - $p_{i1}(t_{end}) = 0$;
    - `// Draw new population`
    - Draw $p_{i0}(t_0)$ from Poisson$(S_0 \cdot \frac{S_i}{S_l})$;
    - **if** $p_{i0}(t_0) > 0$ **then**
      - Add strain i with population $p_{i0}(t_0)$ to the new community
    - ;

S11 Algorithm Method to propose new communities based on their degradation scores from the previous round. Called by S10.
