# Supplementary material for: Novel artificial selection method improves function of simulated microbial communities
Source: PLoS Comput Biol. 2026 Jan 13;22(1):e1013863. doi: 10.1371/journal.pcbi.1013863 (PMC12829962; doi:10.1371/journal.pcbi.1013863)
Supplement: S1 Note — Supplementary methods on the implementation of the ODE model. (PDF) [file pcbi.1013863.s035.pdf]

S1 Note

Supplementary methods describing ODE model

As in the IBM, the ODE model simulates well-mixed batch cultures with nutrients and toxic compounds, extending a previous model [30].

In the model, the population size S_i of each strain i in a community grows in relation to the concentrations of nutrients N_j and decline by the toxic compounds T_k (Fig. S1) by the model parameters in Table S4. Growth, death, nutrient uptake and degradation is described by the following ODE system:

$$\frac{dS_i}{dt} = \left((1 - \sum_k f_{ik}) \rho_i(\mathbf{N}) - \mu_i(\mathbf{T}) \right) S_i \tag{11}$$

$$\frac{dN_j}{dt} = - \sum_i \frac{\rho_i(N_j)}{Y_i} S_i \tag{12}$$

$$\frac{dT_k}{dt} = - T_k \sum_i f_{ik} \delta_i \rho_i(\mathbf{N}) S_i \tag{13}$$

The bold-face $\mathbf{N}$, $\mathbf{T}$ denote the vectors of all nutrients and toxic compounds, respectively. We assume Monod and Hill functions for the per-capita growth and death rates ρ_i, μ_i.

$$\rho_i(\mathbf{N}) = \sum_j r_{ij} \frac{N_j}{N_j + K_N} \tag{14}$$

$$\mu_i(\mathbf{T}) = \sum_k m_{ik} \frac{T_k^2}{T_k^2 + K_T^2} \tag{15}$$

The system of equations (11)–(13) is solved with a standard ODE solver (`dopri5`, [43,46]) for 100 time steps with initial conditions $S_i(t_0) = 100$, $N_j(t_0) = 100$ and $T_k(t_0) = 100$ for all i, j, k.

The investment f_{ik} can mutate to form different strains of the same species. When this happens, we add a new population equation of the type (11) to the ODE system, with the same parameters r_{ij}, m_{ik}, Y_i and d_i as the ancestor but with the modified f_{ik}. To not make the system of equations too large, we have limited the number of strains to 28 per community. We estimate this to be enough since we expect mutants to rapidly replace their ancestral strains if their growth rate is higher, and otherwise disappear rapidly. If there are already 28 strains in a community, then no more mutants are allowed. Otherwise, when communities are propagated to the next round of growth, any surviving strain can have a mutant with probability 0.05. Having chosen which strains i to mutate, we pick one or more traits f_{ik} at random and multiply them by numbers drawn at random from lognormal$(0,\ 0.4)$ and ensure that both the mutated traits f_{ik} and the total investment $f_{i\cdot}$ falls in the $[0,\ 1]$ interval. The mutant receives the same r_{ij}, m_{ik}, Y_i and d_i parameters as its ancestor and is introduced with population size 100, the same as the initial population before the first round of growth. This population size is chosen relatively high, in order to speed up the competition between ancestor and mutant strain.
