# Supplementary material for: Novel artificial selection method improves function of simulated microbial communities
Source: PLoS Comput Biol. 2026 Jan 13;22(1):e1013863. doi: 10.1371/journal.pcbi.1013863 (PMC12829962; doi:10.1371/journal.pcbi.1013863)
Supplement: Attachment — Submitted filename: vessman_guridi_response_to_reviewers.pdf [file pcbi.1013863.s036.pdf]

Response to referees

Manuscript number: PCOMPBIOL-D-25-01028
Title: "Novel artificial selection method improves function of simulated microbial communities"

We are very grateful to all three reviewers for their comments, which we think have greatly helped to clarify and improve the manuscript. Reviewers' comments are written in black below, and our responses in blue font and indented text. Direct changes to the text are underlined here and highlighted in blue in the revised manuscript.

Reviewer #1:

This manuscript proposes a new selection method to improve the functional performance of microbial communities through artificial selection. The key challenges in this area involve ensuring group-level trait inheritance and maintaining sufficient between-group variation. The proposed method, called "disassembly selection (DS)," addresses these challenges by actively increasing between-group variation—achieved through the addition or removal of species in offspring communities. This strategy enables the exploration of a wide range of species combinations, thereby enhancing the likelihood of discovering high-functioning community compositions. The manuscript demonstrates that this method outperforms conventional selection strategies. I enjoyed reading the manuscript and found that the results are both interesting and impactful for the field of microbial community evolution. However, the dynamics of the model system and the underlying mechanisms by which DS outperforms other methods remain somewhat unclear to me. I therefore recommend publication after revision.

We thank the reviewer for the positive feedback and are very glad they see the value of our work. We also appreciate that the mechanisms behind our findings were not very clear and hope that the revised version of the manuscript has improved on this point.

[1] In Fig.2, the rank of degradation score for all of the 32767 possible combinations of ancestral species are shown. I presume that the score may depend on the initial condition. For example, if a single species community starts with only 10 cells while a four-species community starts with 40 cells (10 from each), naturally the latter would have higher total biomass and thus potentially better degradation performance. This could introduce a bias where species-rich communities appear more effective simply due to larger biomass. Has this effect been controlled for when comparing degradation scores and assigning ranks to all possible species combinations?

It's true that we had not tested for the effect of the initial condition (total initial biomass) on degradation score. We have now done this by running additional simulations: We ran all the possible communities (32767) combining 15 species for one round, but now instead of seeding 10 cells per species per tube (such that the total depended on species number), we seeded 150 cells in total per tube divided among the species (if the division of 150/number_species was not exact, the first species had one more cell, etc. until the remainder was 0).

Compared to our original simulations, the best degrading communities in this new datasets were very similar (or even the same for one species set). We calculated the Jaccard distance on the presence or absence of each of the 15 species in the winning community for each species set (1-5) (see Figure 1 below).

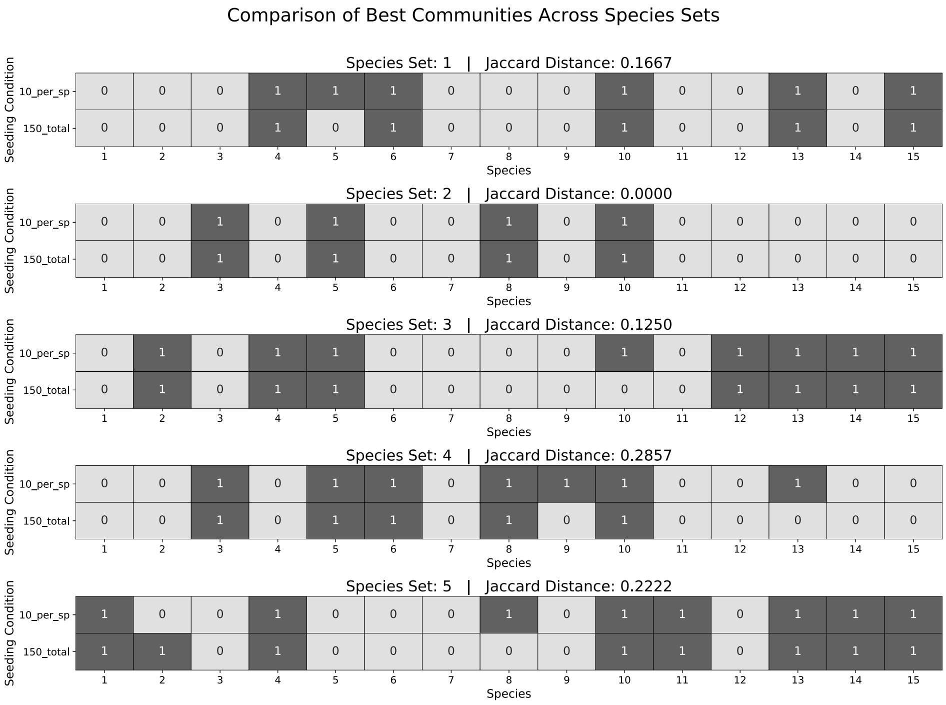

Figure 1 Jaccard distances between the winning communities in each species set after running 32767 species combinations with either 10 cells per species or 150 cells total. Dark grey '1's and light grey '0's indicate presence or absence of each of the 15 species in the winning community. This shows that the two ways of initializing our tubes resulted in very similar winning communities.

To test whether seeding 10 cells per species vs 150 cells total affected the number of species in the winning communities, we compared the number of species in the 10 best degrading communities for each species set and found that this number was significantly lower when we start with 150 cells compared to 10 cells per species (Mann-Whitney U test, P=0.0006). However, the differences were not drastic, in the sense that the best communities were never composed of less than 3 species in either case.

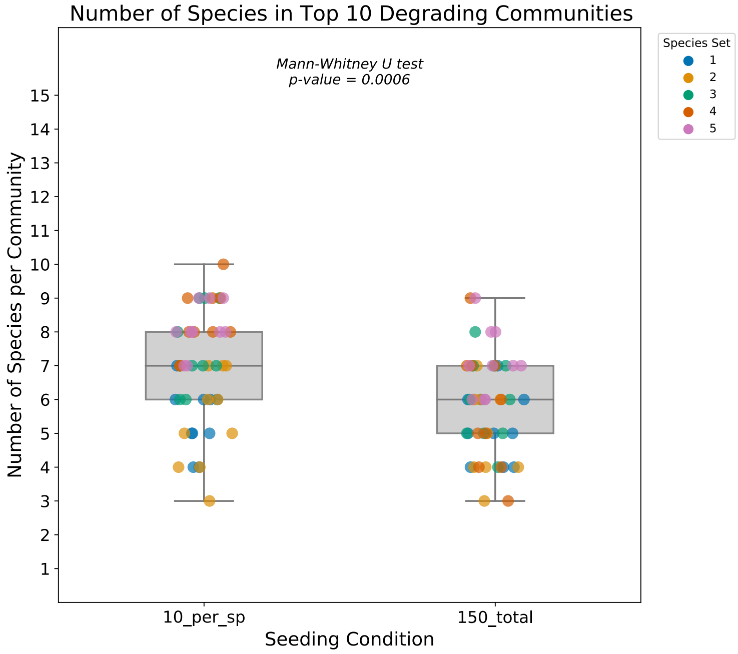

Figure 2 Number of species in each of the winning communities colored by species set in the two seeding conditions. Note that the data in the left boxplot correspond to the yellow points in Fig. 3F in the manuscript (smallest winning community was of size 3 and the largest of size 10 with a median of 7).

We have added this clarification to the Methods section when we describe how we create the inoculum by adding the following:

> "In some simulations, the number of species can vary. When this happens, we inoculate 10 cells of each species thereby changing the total inoculum. We verified that this choice (as opposed to keeping the total constant and partitioning the population among the different species) did not have a strong effect on performance (Fig. S19)."

And have added one supplementary figure with the two above figures as panels.

We also used the ODE model to check whether the inoculum might affect the outcome of the selection algorithm in finding better-degrading communities (Figure 3 below). The short answer is again: yes, it sometimes had an effect (Mann-Whitney U test on each of the five species sets: set 1 $P<0.001$, set 2 $P=0.13$, set 3 $P=0.51$, set 4 $P=0.28$, set 5 $P<0.001$), but there was no clear direction to this effect whereby degradation was higher in the variable inoculum for set 1 (here labelled 21) and lower for set 5 (here 25). We saw no systematic effect in Disassembly random.

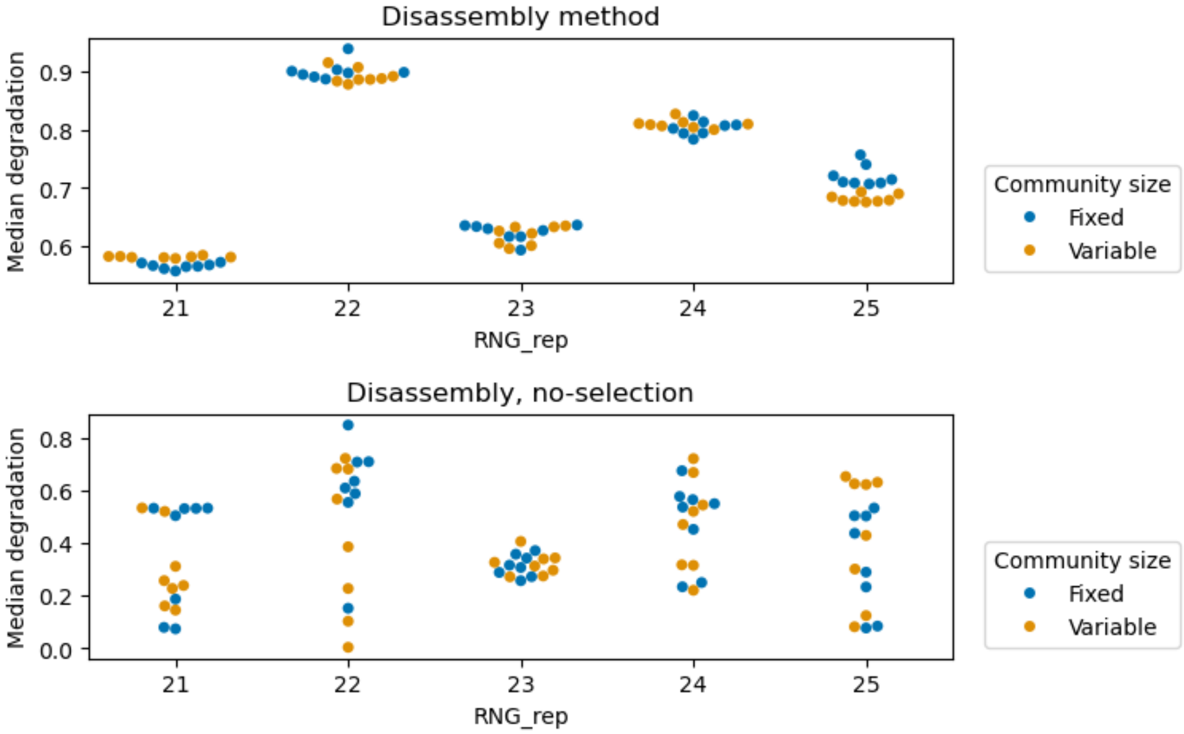

Figure 3 Median degradation of the communities over the last 10 rounds of disassembly selection (top) and random (bottom) if the inoculum was always fixed or variable (a function of the number of species).

[2] What is the composition of the top-ranked (i.e., most optimal) community? What happens if this optimal composition consists of a single species? In such a scenario, the DS method, which emphasizes species diversity, may not be the most effective. Does DS still outperform other methods even when the optimal solution lies in low-diversity or even monoculture communities?

As shown in Figure 2 above, the optimal communities had a median of 6 or 7 species (depending on initial condition), and the smallest optimal community was composed of three species. The strength of DS is not necessarily to increase diveristy, but that it can explore more community compositions, including both larger and smaller communities. It so happens that for this problem, a single species was never the best.

Below (Figure 4) we show a plot of the trajectories of species numbers in our simulations for each selection method which we have added as a new supplementary figure (Figure S6). DS is able to converge to different community sizes depending on the species set. For example, looking at the yellow dots in Figure 2 of this document, we can see that the best communities are often small, and indeed when we run DS, the communities stay small on average. In other words, if we were to apply

DS to another problem where a single species is the best, we see no reason why it would not find this solution.

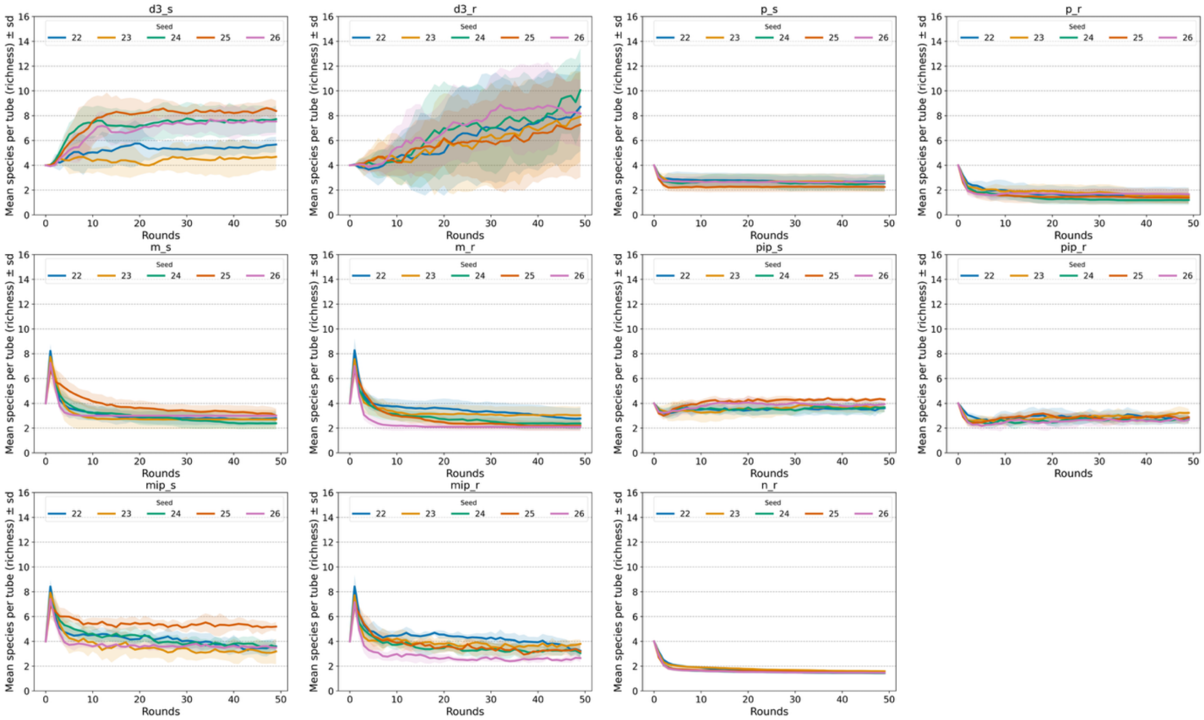

Figure 4 Mean species richness per tube for each species set over rounds of selection for each propagation method. This is including all the tubes, not just the best tubes. We can see that DS (top left panel) is able to maintain diversity, but does not necessarily always go up. PIS can also maintain diversity, but if the optimum is higher, it does not seem to increase as much.

[3] It seems that DS outperforms other methods largely because it supports the persistence of species that are otherwise prone to extinction—by providing them with sufficiently large initial abundances. If this interpretation is correct, I would expect the performance gap between DS and other methods to shrink when the total simulation time is shortened (e.g., by reducing the time step from t=80). Have the authors considered or tested how performance differences vary with the time-scale of selection?

We thank the reviewer for this suggestion. Indeed, it is plausible that more time is needed for the slow growers to establish, adding importance to the run time of each round. We checked for this by varying the time-steps in each round (t=40, 60, 80, 100, 120) with t=80 being the one used previously.

For all propagation methods, we found that the more time they have, the more they degrade (positive correlations between time steps and maximum degradation, all p-values<10^-7, see Figure 5). This is intuitive as they have more time to degrade, but on the other hand, degradation ceases once all nutrients have been consumed. In DS, it appears that we have reached a plateau where we think the communities depleted the nutrients before the end of the 100 or 120 time-steps, such that the additional time does not add much or even decreases performance a bit. We have added a panel to Figure 6 in the manuscript and the full data (Figure 5 here) to the supplement, along with the following text:

> “[..] the number of timesteps per round appears to be close to the optimum at t = 80. When rounds are very short, cells do not have enough time to degrade, but if rounds become too long

and the community is performing well, nutrients are likely depleted around t = 80 such that cells do not benefit from the additional time (Fig. S18).”

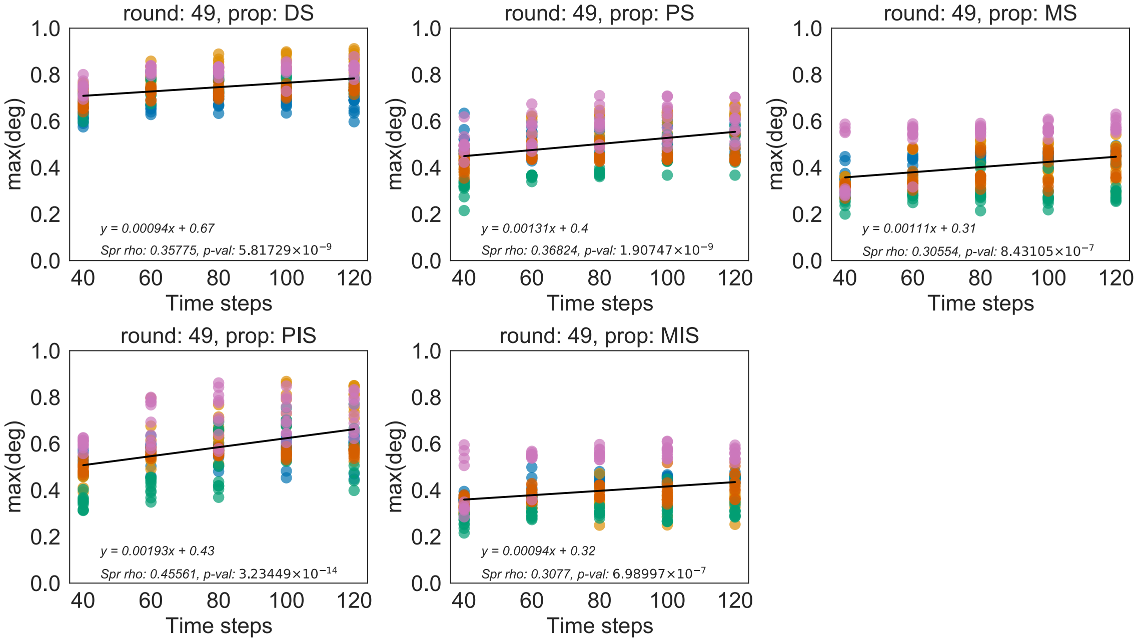

Figure 5 Effect of the number of timesteps in each round on the maximum degradation score.

Reviewer #2: The paper presents a model of microbial population dynamics and toxin degradation, with the goal of selecting for communities with the highest degradation. This is an important and interesting topic, the manuscript is well written and the figures are clear. But the proposed “disassembly method” appears very impractical and I have some more specific comments below.

We are glad the reviwer finds the topic important and the paper to be clear. We also agree that the strong point of the method we proposed is not practicality. Our goal was to use the models to test whether the disassembly method would be worth implementing experimentally. As we had good preliminary data from these simulations when we started the project we went ahead and carried out the experimental test, which was published last year (https://doi.org/10.1038/s41467-024-52190-z). Finalizing the computational work took longer, but we kept the model in line with the experiments, as we find that there are still important lessons to learn from it for future experiments, as outlined in the discussion of this and the experimental manuscript.

1. My main concern is that the key feature of the disassembly method is “controlling the ecological dynamics […] tightly” (ll. 265). This means that (the most “optimal") communities are continuously restarted with equal abundances of all surviving community members, extinct species are re-introduced in at least some of the new communities (if I understand correctly), and there is some additional random re-shuffling of species (“migration”). Maintenance of diversity in this method is thus “enforced”, and it is not surprising that this allows DS to explore more of the “community” space. But more importantly, the communities found in this way quickly degrade as soon as the tight control is removed, losing the advantage gained by the tight ecological control (ll. 261ff). It is thus very questionable how practically relevant this artificial selection method is, as this would require constant monitoring or frequent resetting of the (unstable) community. The authors also acknowledge the problem of instability in the discussion (ll. 351ff).

We agree that this indeed an important issue, and as the reviewer is pointing out have therefore discussed it in the manuscript. But it is one of the lessons we have learned from our simulations, which we think is an important result, even if this means that the DS method does not solve all our problems. We elaborate on how we are applying these lessons to improve the method below and in the manuscript itself.

This is compounded by other practical concerns: Building the repository of species from the highest scoring communities after each round requires an isolation step. Apart from the practical and labour-intensive (also mentioned in ll. 289ff) problem of how to differentiate and isolate the different species (streak plate assay and overnight cultures?), this step presents additional selection pressures unrelated to the task of toxin degradation. First and foremost, in practice this step would probably always require all relevant species to be able to grow in isolation, if only for them to grow up to the necessary densities after isolating single/a few clonal cells. This would seem to preclude the selection/evolution of any sort of cross-feeding or division of labour strategies, where for example some species predominantly invest into toxin degradation while relying on the community for at least some metabolic processes. The presented model does not explicitly include the isolation step or cross-feeding interactions, which is fine for simplicity reasons. But the isolation step is an integral part of the DS method and its potential to actively select against a tighter integration of the community and its function needs to be discussed in some more detail. In particular, because the propagule and migrant pool selection protocols do not require an isolation step and thus do not run into the same potential problem.

Regarding the first point on experimental feasibility, Disassembly Selection is indeed very labor-intensive when implemented in the lab. We have described this in detail in the corresponding experimental paper (https://doi.org/10.1038/s41467-024-52190-z). But we did manage to isolate the species using selective media and plating at each round to separate the species from one another. We would then collect cells from the colonies, freeze them down and then revive them to create new communities in the next round. The experiment yielded interesting results: "The best community found using this approach performed significantly better than the best initial communities (Wilcoxon rank sum test, $p < 0.001$) and 69% better than the community studied in our previous work (Wilcoxon rank sum test, $p = 0.007$, Piccardi et al. 2019)" (from https://doi.org/10.1038/s41467-024-52190-z).

On the second point about dependencies between community members: it is true that in the DS method, all species need to be able to grow alone on some media in the lab – not necessarily under the conditions under which they are evolving, though. For example, if we were to apply community level selection in the absence of amino acids and vitamins, but then isolate each species on rich medium, positive interactions and cross-feeding could still play a role. In our model we include cross-detoxification rather than cross-feeding as positive interactions that can arise between species. Still, the reviewer is correct that in PS and MS, one could begin from natural communities without the need to isolate at all, which we agree would probably favor tighter relationships between species beyond those that can be broken by cultivating on rich medium. We have now added this point in the discussion section:

> "Propagule selection has important advantages that we lose with disassembly selection: (i) resulting communities are ecologically stable and (ii) there is no need to culture individual isolates, allowing for obligate mutualists -- a point we have not explored here."

2. From a more theoretical point of view I have the feeling that as soon as you start isolating species/strains, there are a myriad ways of recombining them for the next round. The proposed DS method is one of those methods that seems to perform better (in some sense) than PS and MS propagation, but why look at this specific method with these specific parameters in the first place? Is there a rational behind this, is it the simplest starting point? More concretely: The authors state that "in the disassembly method, more and better communities can be found by randomly adding and removing species" (ll. 207ff). Maybe I missed this, but is the case of NOT adding and removing species explored (DS without migration)? In other words, how much do the results change if the step of random loss or gain of a species in a subset of communities (ll. 129-130) does not happen? Or if the number of randomly chosen communities (here 5) varies (briefly touched upon in ll. 304, but not discussed?)? I guess this corresponds to changing the speed of exploration of the "community space" in some sense? This would be interesting, especially since the PS and MS methods (no emigration or immigration) are compared to corresponding variants with migration (PIS and MIS, ll. 205ff). In general I think the section exploring different values for some of the key parameters (ll. 287ff) would benefit from some more detailed explanations or discussions. For example, it is just mentioned that "the number of communities receiving an invading species is negatively correlated with degradation". This sounds curious, but what is the reason for or significance of this observation?

We have not tested DS without migration, but we expect this to greatly reduce performance. Essentially, we would be restricting the search space to the 21 species combinatinos that were randomly assembled in the first round, and hoping that one of these is among the best out of 32767 possible species combinations. We only have two ways of improving on these 21 communities in DS: through within-species mutation and through migration. This is because we are controlling everything else: repeating the simulation for the same species combination several times will always give approximately the same performance (there is some stochasticity in the model of course). We showed that mutations did not improve performance in DS (Fig. 5 in the manuscript). So if we set migration to zero the performance should be approximately what it is at round 0. In other words, we think we would end up with similar problems as PS and MS.

Beyond setting it to zero, it's true that we observe this negative correlation between performance and the number of communities to receive/lose a species. Our understanding is that too many changes to a community can be disruptive, quickly destroying successful solutions. We now discuss this and other parameter changes more extensively in the results section:

> "The number of communities receiving an invading species is negatively correlated with degradation ($\rho = -0.29$, $p < 10^{-9}$). This is likely because too frequent invasions make it difficult to maintain high-performing species combinations. On the other hand, invasions cannot be reduced to 0, as DS relies on mixing species to explore the search space. The optimum appears then to lie in introducing species with a small, positive frequency (Fig. S14)."

3. ll. 82 and ll. 565: Does fixing the initial number of cells per species really correspond to maintaining heritability? While this might ensure persistence of species (which is part of heritability, I guess?), it also resets the final community state of the previous round (i.e. destroying a heritable(?) "trait" of the community). More generally, what does heritability actually mean in this context? Maintaining the presence/absence of species? I think the authors briefly touch upon this in ll. 347, mentioning that they are "sidestepping the issue of heritability". Here I was also wondering if the suggestion to "achieve individuality first, and selection [for degradation] second" is somewhat arguing against the DS method and more in favor of e.g. the PS method? Since the PS method lets the ecological dynamics stabilize and then selection for degradation could act on the resulting (stable but still "mutating") community?

Thank you for this comment, which has helped us clarify what we mean by heritability in our paper. We have changed the following text in the introduction (altered text is underlined):

> "<u>Heritability – 'the regression slope of average offspring phenotype on average parental phenotype' (Bourrat, 2022) -- is one of the requirements for evolution by natural selection, but groups of organisms naturally have lower heritability compared to canonical individuals (Goodnight, 2000).</u> The problem arises because a single community-level 'generation', which we call a 'round of selection' to avoid confusion, can comprise several generations of cells, each belonging to different genotypes (i.e. species or strains). Since the genotypes all reproduce at varying rates, their relative abundances can change during one round of community growth and over subsequent rounds. Because community <u>function</u> depends on the traits of all of its constituent genotypes and their proportions, an 'offspring' community may not resemble its 'parent' (Goodnight, 2000; Williams & Lenton, 2007; Xie & Shou, 2019)."

In any microbial culture with defined species composition, heritability of its function (here toxin degradation) can be reduced through mutations within each of the species, stochasticity in the system, and changes in the relative abundance of each community member. Unless the communities are ecologically stable, the latter factor tends to have the biggest effect on reducing functional heritability. Xie & Shou, 2019 showed that for community-level selection to succeed, one needs to find a way to increase heritability. One way to do so is to wait until the community stabilizes, such that the relative abundances stop changing (indeed, this is a strength of the PS method). The alternative is to "force" heritability to remain high by always beginning each culture with the same relative abundance. This is what we have done and what we meant by "sidestepping the issue of heritability". In other words, we are leaving it for future work, and discuss how to do so in the discussion section. We have now tried to clarify this in the discussion:

> "As in (10), we sidestep the issue of heritability and ecological stability in the disassembly method by always re-assembling communities in a fixed abundance and equal species proportions. This allows community dynamics to unfold in almost the same way in each round during selection, such that offspring communities resemble their parents."

One of the reviewer's questions was why heritability is not changing from one generation to the next in our model. According to our definition of heritability what matters is the offspring phenotype relative to that of its parent, where we compare offspring and parent phenotype (community function) at the time when they have reached adulthood, i.e. at the end of a round of growth. So reducing the population size through dilution is not what we would call a reduction in heritability, but is simply part of the lifecycle of a community.

The next question is how do we solve the heritability problem in the long-term, besides just "side-stepping it as we do here". Can we have the best of both worlds? The heritability of PS once communities have stabilized and the generation of variance by DS? In more recent work, we are trying to understand what makes communities more ecologically stable such that their performance does not decline over time once "ecological control" is relaxed (our recently published opinion piece: https://doi.org/10.1016/j.mib.2025.102596). What we propose is "individuality first, selection second". Our idea is to first establish the properties that allow communities to be stable, characterize different species combinations based on these properties and then overlay that on our disassembly selection such that when new communities are composed (though migration), that combinations with higher individuality (heritability, stability, etc.) would be favored. For example, one would only add a species that has a complementary function and where we can be confident that ecological stability can be maintained, or remove a species that would not lead to ecological collapse or reduce functional coverage. We have tried to clarify all this in the discussion section (altered text is underlined):

> "Another solution could be to restrict our search space towards communities that have higher heritability, i.e. communities that behave more like "evolutionary individuals" (38). If we could accurately predict which species combinations are ecologically stable based on species' properties, we could restrict the algorithm to only considering those combinations. We would thereby aim to achieve individuality first, and selection second, increasing the likelihood that the winning community would be one that does not lose its properties after the end of the selection experiment (38)."

4. I was somewhat confused by the paragraph ll. 362 in the discussion. If one removes the isolation and disassembly step, what sort of selection method am I left with? Some form of PS or MS, depending on how I put together the new round of selection?

The isolation and disassembly step were mainly done such that within-species evolution could occur during community selection. But because within-species evolution only leads to reduced degradation (Fig. 5A of the current manuscript) with no significant effect on community performance (Fig. 5C), we are instead proposing to develop a new selection method, where we would work with ancestral strains and use the algorithm presented in this manuscript as a search algorithm to find the best species combinations of ancestral strains. This also allows us to increase the species pool, which Fig. 6C shows to be one of the most important factors. This is not PS or MS, because the strength of this revised DS (which should not be called disassembly selection, as there is no disassembly) would be to explore the space of species combinations thoroughly, which we are arguing is the weakness of PS and MS. We have added a sentence (underlined below) to clarify this point in the discussion:

> "This means that experientall, one need only work with ancestral species, avoiding the need to disassemble communities into their individual members. At each round then we would select the best communities, and rebuild them from the *ancestral* freezer collection after changing their composition with a given probability by removing or introducing species."

We hope that the reviewer thereby appreciates that while the problem is multi-faceted, the simulations presented in the current manuscript have taught us a lot about community-level selection and how to move forward. We think that other readers of our paper can equally benefit from our experience, even if this paper does not contain all the solutions.

5. IBM vs. ODE model: If both models show similar results, why present the IBM model and its results? An ODE model seems more straightforward and potentially easier to reproduce? Can the authors give a rational for why to present the IBM?

Historically, we began the project with the ODE model but had difficulties implementing mutations: each time a mutation occurs, one needs to increase the number of differential equations in the system. This is indeed how we implemented it, but to not make the system of equations unreasonably large, we limited the number of strains to 28 per community. If there are already 28 strains in a community, no more mutants were allowed. While this is arguably acceptable, the IBM gave us more flexibility and we could avoid such issues. For a similar reason, we found it challenging to implement Migrant Pool Selection in the ODEs, as species and strain numbers were more likely to increase beyond these 28 slots and it seemed unreasonable to be capping diversity. We have now added a brief explanation of this issue in the Methods section to justify our choice:

> "We chose to describe the IBM in the main text and the ODE model in supplementary methods, as the ODE model obliged us to fix an upper limit to the number of strains in a community (number of differential equations), making it difficult to implement mutations and the migrant pool method. The ODE model therefore only recapitulates a subset of the results."
