# Supplementary material for: Novel artificial selection method improves function of simulated microbial communities
Source: PLoS Comput Biol. 2026 Jan 13;22(1):e1013863. doi: 10.1371/journal.pcbi.1013863 (PMC12829962; doi:10.1371/journal.pcbi.1013863)
Supplement: Attachment — Submitted filename: Vessman_Guridi_Response_to_Reviewers_auresp_2.pdf [file pcbi.1013863.s037.pdf]

Response to referees

Manuscript number: PCOMPBIOL-D-25-01028R1
Title: "Novel artificial selection method improves function of simulated microbial communities"

We are very grateful to the reviewers for their additional comments. Reviewers' comments are written in black below, and our responses in blue font and indented text. Direct changes to the text are underlined here and highlighted in blue in the revised manuscript.

Reviewer #1:

The authors have addressed all of my comments satisfactorily, and I now recommend this manuscript for publication.

We would like to thank the reviewer for taking the time to review our revised manuscript and are happy that the changes were satisfactory.

Reviewer #2:

I thank the authors for their detailed responses and am going for "accept" at this point.

I still do not buy into "favoring" the IBM model over the ODE model, at least not based on the arguments the authors have put forward in this specific case. And now there seems to be a little contradiction between "both models showed very similar results" (ll. 72) and the newly added "The ODE model therefore only recapitulates a subset of the results" (ll. 419).

Generally I think the more concise, tractable and reproducible definition of a model in terms of a system of differential equations far outweighs any, often just perceived, drawbacks. The system could probably be written down in just a few lines (even considering the complication of stochastic mutations, discrete transfers/reassemblies and the resulting hybrid dynamical system) and computationally it is no (big) problem to solve ODE systems with hundreds or even thousands of state variable/equations. And arguably this shouldn't ever really be necessary for these kinds of problems anyway (only a (potentially large, but still relatively smallish) subset of all possible species/mutants will ever have non-zero densities over a given timespan; one can reasonably discretize the "mutant space" to reduce the number of all possible mutants; one can "cheat" a bit by removing mutants if densities fall below a certain extinction threshold, i.e. resizing the system dynamically; etc.).

The IBM model on the other hand is effectively "defined" by, as far as I can see, thousands of lines of code. Realistically this makes it impossible, or at least very impractical, to trace what really is going on, even with the (nice!) inline comments and ten page documentation. I'd also be surprised if this is computationally more efficient than numerically solving a (very) large ODE system.

Again, notwithstanding my above "more a comment than a question" kind of comment, I am ok with recommending "accept".

We would like to thank the reviewer for taking the time to review the revised manuscript and are glad they agree that it is now acceptable to be published. And we do see the point of why an ODE model might be favorable. That said, we don't really see the utility of switching everything around at this point (modifying the figures such that the main ones are based on the ODE and the supplementary ones on the IBM). Of note, in the original version of the mansucript (that was reviewed at another journal), we had both models in all the figures side-by-side and the reviewers complained that it was too much information. And perhaps there would be a way to implement the migrant pool method using the ODE model, but again at this point we are not sure it would bring much to invest into modifying the implementation of the ODE model. The editor also made a point that in a computational biology journal it maybe makes sense to put the IBM at the forefront.

In any case we have clarified the choice in the text with more nuance. In the results section when we introduce the models we write the following (added sentence is underlined):

> “To make sure that our results are not biased by the type of model we chose or its exact implementation, we developed an individual-based model (IBM) and a model based on ordinary differential equations (ODE). As both models showed similar results, we present the results of the IBM in the main text and leave the ODE model as a comparison in the supplement. Each model has its strengths and weaknesses – the IBM being more flexible to implement experimental details and the ODE being simpler to reproduce and understand – but we chose to focus on one for conciseness and leave it to the reader to explore the other if preferred.”

We hope this addition clarifies our choice.
